# Non-histone protein L-lactylation in cancer: a metabolic switch for immune evasion and therapeutic resistance

**DOI:** 10.1186/s12943-026-02759-7

**Published:** 2026-08-01

**Authors:** Yingli Guo, Lanyang Gao, Shunda Li, Jicun Chen, Wenguang Fu, Xuelei Ma

**Affiliations:** 1 Department of Biotherapy, Cancer Center and State Key Laboratory of Biotherapy, West China Hospital, Sichuan University, Chengdu, Sichuan China; 2 Academician (Expert) Workstation of Sichuan Province, Fundamental and Clinical Research on Mental Disorders Key Laboratory of Luzhou, The Affiliated Hospital, Southwest Medical University, Luzhou, 646000 China

**Keywords:** L-lactylation, Non-histone proteins, Cancer metabolism, Immune evasion, Therapeutic resistance, Tumor microenvironment

## Abstract

Initially characterized as an epigenetic marker for transcriptional regulation, lysine lactylation is now recognized as a pervasive posttranslational modification with extensive functions beyond those associated with chromatin. Recent methodological advances in the resolution of stereoisomeric dynamics have established L-lactylation as the predominant glycolysis-derived functional form, providing a metabolic switch that couples glycolytic flux to oncogenic signaling. Here, we provide a comprehensive overview of non-histone L-lactylation in cancer biology. We define the spatially compartmentalized catalytic network governing this process, detailing how nuclear EP300/CBP, cytosolic AARS1, and mitochondrial AARS2 mediate stereospecific targeted modifications. At the molecular level, non-histone L-lactylation alters protein biophysics via charge neutralization, steric hindrance, and interface remodeling. These physicochemical alterations govern fundamental enzymatic kinetics, complex assembly, subcellular trafficking, and proteasomal degradation processes. Through primarily transcription-independent mechanisms, L-lactylation enables cancer cells to sustain metabolic flexibility, promote the repair of damaged DNA, and foster an immunosuppressive tumor microenvironment. Because tumors exploit these regulatory networks to drive adaptive resistance across diverse therapeutic modalities, we examine current strategies for pharmacological intervention. Finally, we highlight critical unresolved questions in this field. Notably, the identification of lactylation-specific readers and the development of stereoisomer-resolved chemobiological tools will be essential to fully leverage this metabolism–modification axis for cancer therapy.

**Supplementary Information:**

The online version contains supplementary material available at https://doi.org/10.1186/s12943-026-02759-7.

## Introduction

Since the initial discovery of histone lysine lactylation (histone Kla) in 2019, this modification has been widely defined as a metabolic sensor linking the Warburg effect to epigenetic regulation [1]. While subsequent research has led to the generation of extensive literature reports and multiple systematic reviews detailing its role in the control of gene expression, this chromatin-centric perspective captures only a fraction of the biological utility of lactylation.

Recent high-resolution proteomic mapping revealed that the majority of L-lactylation sites are present on non-histone substrates [2–5]. Notably, advanced orthogonal techniques have recently elucidated the stereochemistry of this modification, indicating that glycolysis-derived L-lactylation represents the predominant functional form [6]. Lactylation occurs widely among cytoplasmic and mitochondrial proteins with various functions, including metabolic enzymes, signal transducers, RNA-binding proteins, and cytoskeletal components [7–10]. In diverse tumor models, such as hepatocellular carcinoma (HCC), gastric cancer, and prostate cancer models, numerous non-histone L-lactylation sites have been identified and confirmed to regulate protein-level processes, including metabolic enzyme activity, protein–protein interactions (PPIs), protein stability, and subcellular localization [5, 11–13].

This broad structural distribution implies that focusing exclusively on transcriptional regulation restricts the functional scope of L-lactylation in cancer biology. Histone L-lactylation modulates gene expression by altering chromatin states; consequently, its functions manifest at the protein level only after multiple transcription and translation steps [14, 15]. In contrast, the direct targets of non-histone L-lactylation are effector proteins, enabling functional adjustments through changes in enzyme activity, interaction interfaces, or targeted evasion of proteasomal degradation [16–18]. This mode bypasses transcriptional and translational delays and provides cancer cells with a more immediate mechanism for coupling the metabolic state to protein function. Such a capacity is likely to be particularly critical during acute stress, such as chemotherapy, hypoxia, or nutrient deprivation. Notably, however, certain non-histone L-lactylation events exert downstream effects through transcriptional mechanisms (e.g., when lactylated non-histone proteins acquire transcriptional activation capacity). Thus, “non-histone” and “nontranscriptional” are not entirely synonymous, and this distinction is explicitly noted in subsequent sections.

Although non-histone L-lactylation has been documented in lung [19], liver [20], gastric [21], and pancreatic cancers [22] and its functions in metabolic reprogramming, DNA damage repair (DDR), immune evasion, and therapeutic resistance increasingly being revealed, systematic synthesis and critical evaluation methods are lacking in this field, which currently faces significant conceptual and methodological bottlenecks. The known substrates, sites, and functional relationships remain scattered across isolated reports without a unifying framework. Furthermore, evidentiary rigor varies considerably, with many studies relying on correlative associations rather than causal validation or failing to resolve the stereoisomeric configuration of the modification. Consequently, the trajectory from fundamental biochemical findings to clinical translation remains poorly defined.

This review systematically integrates reported cancer-associated non-histone L-lactylation events. Beginning with the spatial logistics of catalytic mechanisms and the molecular basis of functional regulation, we delineate how these modifications influence cancer cell metabolic adaptation, genome stability, and microenvironment remodeling. On this mechanistic and functional basis, we further examine the convergent processes driving adaptive resistance across distinct therapeutic contexts. Building on these insights, we discuss potential clinical intervention strategies, including combinatorial approaches utilizing dietary restriction and immunotherapy. Our aim is to provide an organized compilation of the current knowledge on cancer-associated non-histone L-lactylation and to assess its implications in therapeutic resistance (Table S1). Unlike recent reviews that remain histone-centric or treat non-histone substrates briefly, this review is organized by catalytic compartment (CoA- vs. AARS/AMP-dependent) rather than tumor type, applies explicit stereochemical/methodological evidence grading, and frames non-histone lactylation as a convergent adaptive stress response driving resistance.

## Methodological considerations and catalytic mechanisms

### Methodological limitations in lactylation research

**Fig. 1:**
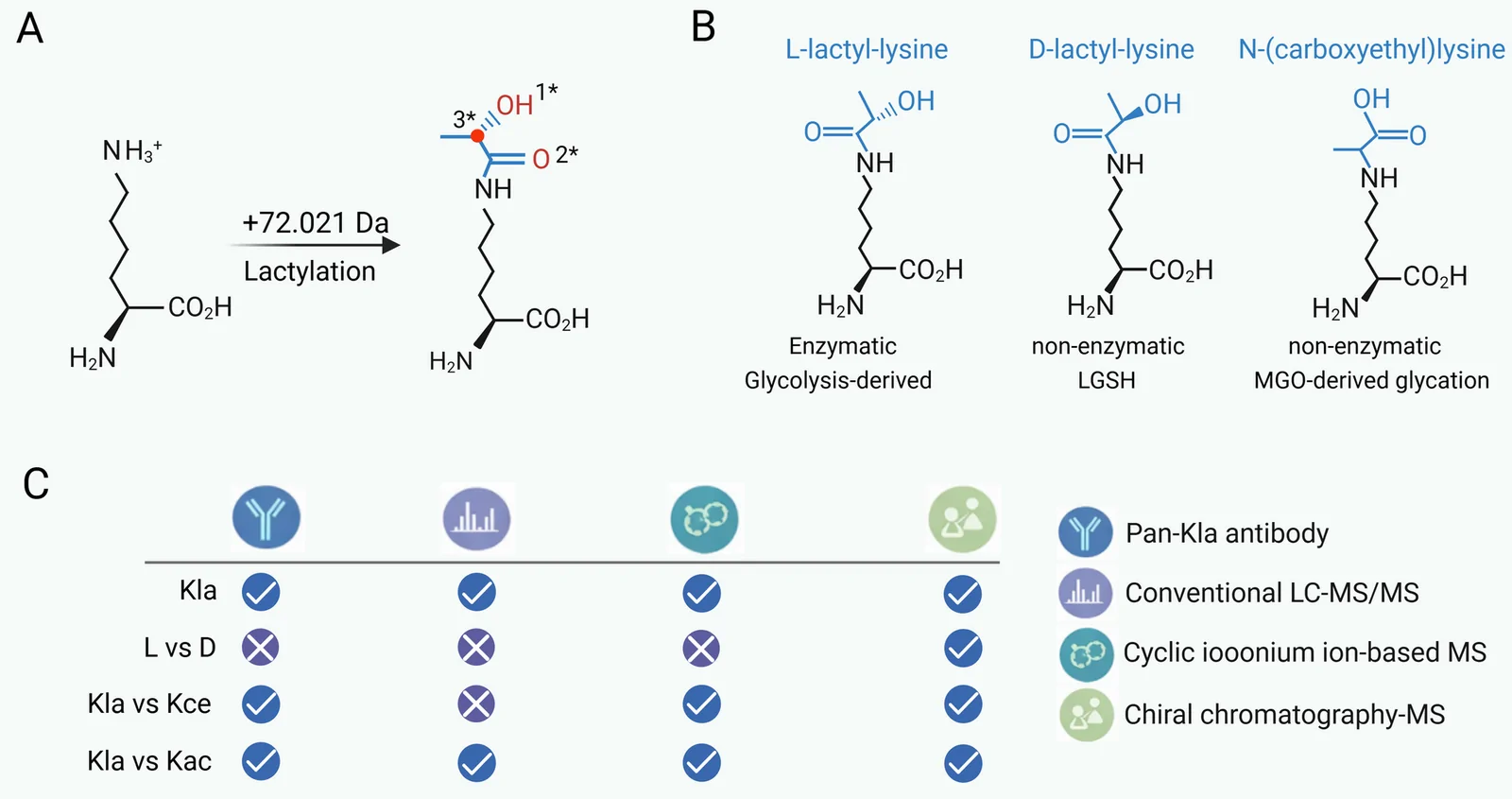
Biochemical properties and stereochemical resolution of lysine L-lactylation. **A **Schematic of the lysine L-lactylation reaction, detailing the + 72.021 Da mass shift resulting from L-lactyl conjugation. **B** Structures comparisons, formation modes and biological sources of L-Kla, D-Kla and N-(carboxyethyl)lysine (Kce). **C** Matrix evaluating the performance of four mainstream detection methods for identifying L-Kla and discriminating it from constitutional isomers and enantiomers. Kac, Lys acetylation; Kce, lysine N-ε-carboxyethyl; Kl-la, Lys L-lactylation; 1* β-hydroxyl group; 2* steric bulk relative to acetyl group; 3* lactyl moiety chiral carbon

Three lactylated Lys isomers have been reported: Lys L-lactylation (K_L−la_), Lys D-lactylation (K_D−la_) and N-ε-(carboxyethyl)-lysine (Kce) (Fig. 1a and b) [23, 24]. Accurate identification and functional validation of these modifications are currently limited by three major technical challenges.

A fundamental analytical barrier involves the stereochemical resolution of L- and D-lactate. Because these enantiomers possess identical masses and chemical formulas, conventional antibody enrichment and tandem mass spectrometry (MS/MS) have suggested to be insufficient to distinguish between them [25, 26]. Their discrimination typically requires chiral chromatography, chemical derivatization, or specialized detection strategies [15, 27, 28]. This limitation has led to widespread analytical ambiguity in the literature regarding L- and D-lactylation (Fig. 1c) [24]. Biologically, this distinction is critical: L-lactate is primarily produced via glycolysis and associated with the Warburg effect [27, 29, 30], whereas D-lactate arises from the detoxification of methylglyoxal and accumulates under carbonyl stress [23, 27, 31, 32]. Consequently, studies emphasizing glycolysis-linked L-lactylation are highlighted in this review, while those lacking stereochemical or metabolic validation are annotated accordingly.

Compounding this analytical challenge is the uncertain specificity of commonly used pan-Kla antibodies, which often exhibit potential cross-reactivity to structurally related modifications such as acetylation. Although MS/MS approaches have improved site-specific detection by utilizing diagnostic cyclic iminium ions [33], many studies still rely on antibody-enriched MS workflows, which may generate false-positive results (Fig. 1c).

At the functional level, traditional site-directed mutagenesis approaches introduce significant confounding variables. Functional validation often employs lysine-to-arginine (K→R) mutations to prevent modification; however, this approach eliminates all lysine-based modifications, including acetylation and ubiquitination, at the site of the mutation, making it difficult to attribute phenotypic effects to lactylation alone [34]. Conversely, lysine-to-glutamine (K→Q) substitutions can partially mimic charge neutralization but fail to recapitulate the steric bulk and hydrogen-bonding properties of native lactylation [34, 35]. Moving forward, chemobiological strategies, particularly genetic code expansion for the site-specific incorporation of lactyl-lysine, will be essential for orthogonal validation, providing a more accurate representation of the endogenous state of modification.

### Spatial compartmentalization of the catalytic network: The biochemical dichotomy

The non-histone L-lactylome is governed by a highly organized catalytic network. The classification of this network is defined not only by physical organelle boundaries but also fundamentally by two disparate enzymatic strategies: CoA-dependent and CoA-independent (AMP-dependent) mechanisms.

#### CoA-dependent mechanisms: Nuclear dominance with cytoplasmic shuttling

**Fig. 2:**
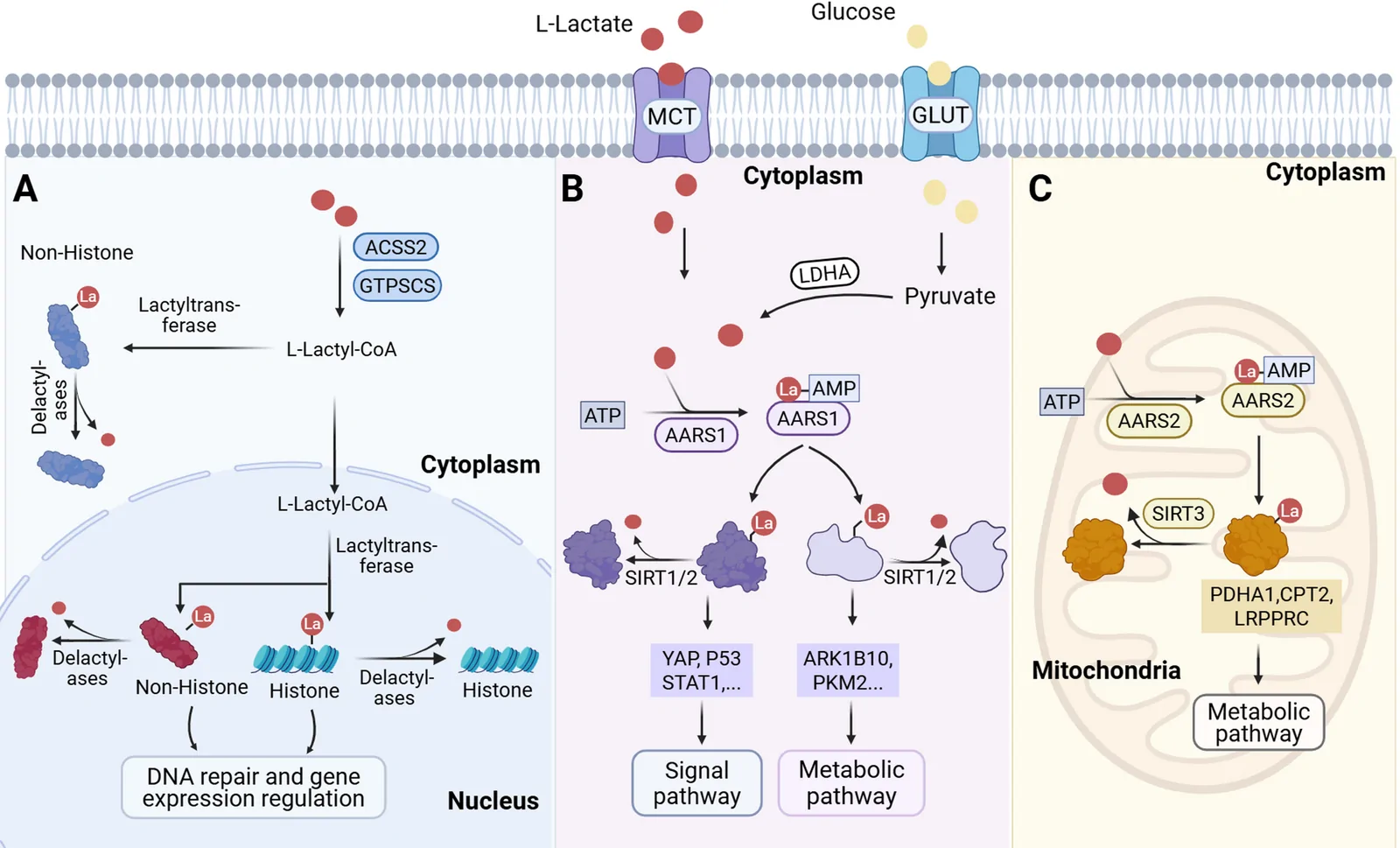
Spatial compartmentalization of the L-lactylation catalytic network in cancer cells. **A** L-lactylation in the cytoplasm and nucleus relies primarily on an acyl-CoA-dependent pathway. L-lactyl-CoA is synthesized by ACSS2/GTPSCS, and then transferred to histone and non-histone proteins by lactyltransferases. Delactylases reversibly regulate this process, which is involved in DNA repair and gene expression regulation. **B** Cytoplasmic L-lactylation is catalyzed by AARS1 via an AMP-dependent route with an L-lactyl-AMP intermediate. SIRT1/2 function as the principal cytosolic de-L-lactylases, modifying substrates related to signaling and metabolic pathways to regulate downstream cascades. **C** Mitochondrial L-lactylation is executed by AARS2 utilizing an AMP-dependent mechanism. This modifies core enzymes of mitochondrial metabolic pathways, and the mitochondrial deacetylase SIRT3 maintains the dynamic reversibility of this process

Canonical L-lactylation relies on L-lactyl-CoA as the high-energy acyl donor and is executed primarily by histone acetyltransferase (HAT) enzyme families (Fig. 2a and b). Among them, EP300 and CBP (KAT3A) are the most extensively validated writers that target a wide array of nuclear non-histone proteins. Their activity is highly dynamic; under metabolic stress, EP300/CBP can translocate to the cytoplasm to modify extranuclear substrates. Documented nuclear targets include transcriptional regulators (e.g., ABCF1 [36] and NUSAP1 [37]), RNA-binding proteins (e.g., HNRNPA1 [38, 39], NCL [40], and METTL3 [41, 42]), DNA repair factors (e.g., MRE11 [43, 44]), and nuclear phase separation modulators (e.g., YTHDC1 [45] and NMNAT1 [46]).

Other HAT families also contribute to CoA-dependent lactylation. As a member of the GNAT family, HAT1 undergoes K15 autolactylation to increase its activity, promoting the lactylation of RPA1 at multiple sites (K88 and K163) [47, 48]. GCN5 catalyzes both XLF K288 lactylation, expanding the Ku70/80 interaction surface to facilitate DNA repair [49], and RCC2 K124 lactylation, recruiting SERBP1 to stabilize target mRNAs [50]. As a member of the MYST family, TIP60 catalyzes NBS1 K388 lactylation, which is critical for stable MRN complex assembly [17].

#### CoA-independent/AMP-dependent mechanisms: Compartment-resident writers

An alternative catalytic route operates independent of L-lactyl-CoA, instead utilizing L-lactyl-adenylate (L-lactyl-AMP) intermediates [51]. The AARS family exemplifies this strategy (Fig. 2b and c). Cytoplasmic AARS1 regulates glycolytic enzyme activity, cytoskeletal remodeling, autophagy, and the stability or activity of cytosolic immune sensors [52–56]. Nuclear AARS1 also contributes to DNA repair, transcription factor inhibition, epigenetic modifications, and posttranscriptional RNA regulation, including m6A modifications and alternative polyadenylation [18, 21, 57–60]. In contrast, AARS2, which is localized predominantly in the mitochondria, modulates energy metabolism and, under hypoxic stress, alters cellular metabolic profiles via the lactylation of mitochondrial enzymes [8]. Furthermore, cytoplasmic AARS2 stabilizes RNA-binding proteins [9] and can be recruited to nuclear complexes to facilitate m6A RNA modifications [21].

Together, these two enzymatic strategies, the CoA-dependent nuclear-dominant and CoA-independent compartment-specific pathways, define a spatially and mechanistically complex lactylation network. This organization ensures that lactylation precisely tunes protein function across diverse subcellular compartments in cancer cells in response to metabolic and environmental cues.

### Spatiotemporal specificity of erasers and the challenge of decoupling

The biological adaptability of non-histone lactylation is predicated on its reversibility, a process mediated by a network of erasers with distinct subcellular distributions and substrate preferences (Fig. 2).

Zinc-dependent histone deacetylases (HDACs) serve as the predominant erasers of the non-histone L-lactylome. HDAC1–3 collectively serve as key delactylases, with HDAC1 and HDAC2 targeting both nuclear and cytoplasmic substrates, including transcription factors [36, 42, 59, 61], RNA-processing proteins [39, 46, 58] and signaling molecules [56, 62, 63]. Conversely, HDAC3 acts predominantly in the nucleus to regulate RNA modifications, transcription, and DNA repair [17, 36, 45, 64]. Notably, the activity of HDAC1 itself can be modulated by lactylation [65, 66].

Sirtuin-mediated de-L-lactylation is strictly dependent on NAD⁺, linking the cellular redox state to lactylation turnover. SIRT1 acts primarily on cytoplasmic, lysosomal, and shuttling proteins, regulating stemness, autophagy, and pyroptosis [55, 67, 68]. SIRT2 exerts negative feedback in the nucleus, influencing m6A/m5C RNA modifications and DNA repair [21, 69, 70] and regulating cytoplasmic glycolytic enzymes (e.g., LDHA) and NHEJ components (e.g., FEN1) [52]. SIRT3 is confined to mitochondria and selectively targets core enzymes involved in the TCA cycle and lipid metabolism [8, 71, 72].

Distinguishing de-L-lactylation from deacetylation remains a critical biochemical challenge, as most HDACs and sirtuins possess dual activity. Consequently, the phenotypic effects attributed to de-L-lactylation may reflect collateral deacetylation. Future research should prioritize the development of catalytic mutants or allosteric inhibitors that selectively target the lactyl-lysine pocket, enabling precise dissection of the independent biological roles of lactylation and acetylation.

## Molecular basis of functional regulation: How L-lactylation alters protein function

Lysine L-lactylation dynamically integrates cellular metabolic status with diverse regulatory processes. The mechanistic principles summarized in this section are derived predominantly from studies in cancer cells or tumor models, and may not necessarily generalize to normal physiology. The addition of an L-lactyl moiety alters the local physicochemical landscape of the target protein by neutralizing the positive charge of the lysine residue, introducing significant steric bulk, and providing a β-hydroxyl group capable of forming new hydrogen bonds. These primary biochemical alterations cause changes in protein function through multiple interrelated mechanisms, including the modulation of enzymatic activity, protein‒protein interactions (PPIs), proteostasis, subcellular localization, nucleic acid binding, and liquid‒liquid phase separation (LLPS). Importantly, these mechanisms are not mutually exclusive; a single L-lactylation event can simultaneously affect multiple functional axes to drive complex biological outcomes.

### Regulation of enzymatic activity

**Fig. 3:**
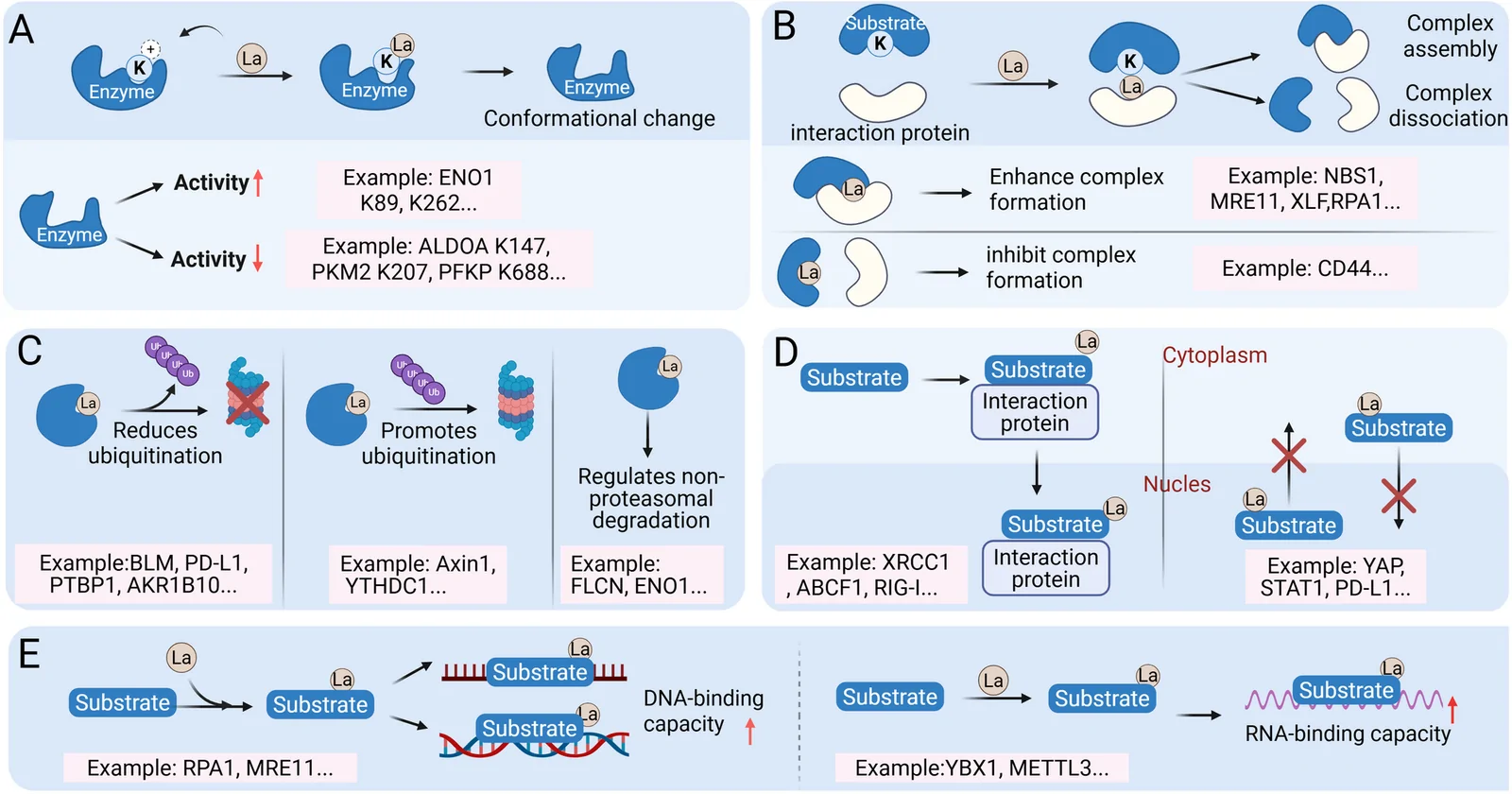
Molecular basis of non-histone L-lactylation in regulating protein function. **A** L-lactylation induces conformational changes of enzymes, and exerts bidirectional regulation on enzymatic activity (upregulation or downregulation). **B** L-lactylation bidirectionally regulates protein-protein interactions, which can either promote complex assembly and formation, or induce complex dissociation and inhibit interaction. **C** Targeted evasion of proteasomal degradation (e.g., PD-L1 stabilization) via competitive interference with the ubiquitin-proteasome system, and additionally modulates non-proteasomal degradation of target proteins. **D** Regulation of subcellular trafficking and spatial compartmentalization, including facilitating nuclear import or blocking nuclear translocation of modified proteins. **E** Modulation of DNA/RNA binding affinities to govern genome maintenance and epitranscriptomic programs

L-lactylation modulates the activities and oligomeric states of key metabolic enzymes primarily through steric occlusion and electrostatic perturbation (Fig. 3a). For example, K147 L-lactylation of aldolase A (ALDOA) blocks the catalytic core, providing negative feedback to prevent excessive lactate accumulation [7, 33, 73, 74]. Beyond direct active site interference, L-lactylation also dictates enzyme oligomerization. For example, K207 L-lactylation disrupts the formation of pyruvate kinase M2 (PKM2) tetramers, promoting dimeric conformations that divert glycolytic intermediates toward anabolic pathways [75]. Furthermore, outcomes are highly dependent on the specific lysine residue modified. In phosphofructokinase, platelet type (PFKP), site-specific L-lactylation produces opposing outcomes: K688 lactylation attenuates enzymatic activity, whereas K392 lactylation enhances glycolytic flux [7, 76, 77]. These examples illustrate that L-lactylation functions as a highly precise allosteric and structural regulator, allowing cells to dynamically balance energy production, biosynthesis, and metabolic feedback.

### Modulation of protein‒protein interactions (PPIs)

Lysine L-lactylation modulates protein‒protein interactions (PPIs) by reshaping electrostatic complementarity and introducing steric bulk into interface regions (Fig. 3b) [78]. By neutralizing the positive charge of lysine, L-lactylation can either promote or inhibit complex assembly in a dynamically reversible manner, coordinated by writers (e.g., EP300/CBP) and erasers (e.g., HDAC1-3) [17, 78, 79]. Mechanistically, lactylation weakens salt bridges [80, 81], adjusts the surface electrostatic potential to fine-tune interface complementarity [17, 44, 82], and generates steric hindrance or competitive binding sites [83–85]. Functionally, L-lactylation can act as a molecular glue: TIP60-mediated K388 L-lactylation of NBS1 promotes the assembly of the MRN complex and the recruitment of homologous recombination (HR) repair proteins [17]. Similarly, K288 L-lactylation of XLF enhances its interaction with the Ku70/Ku80 heterodimer, facilitating nonhomologous end joining (NHEJ) [49], and multisite L-lactylation of RPA1 by HAT1 strengthens its binding to single-stranded DNA and the MRN complex, supporting HR repair [48]. Conversely, L-lactylation inhibits PPIs by sterically blocking interaction surfaces. K158 L-lactylation of CD44 impairs ligand engagement, leading to T-cell exhaustion [86]. Collectively, these studies establish L-lactylation as a potent modulator of PPIs, capable of both stabilizing protein assemblies and inhibiting their formation to regulate cellular signaling and genome maintenance.

### Regulation of protein stability

Targeted evasion of proteasomal degradation has emerged as a major mechanism through which L-lactylation modulates tumor cell physiology (Fig. 3c). Mechanistically, L-lactylation intrinsically interferes with ubiquitin-dependent degradation by competing for targeted lysine residues, altering E3 ligase recognition motifs, or masking degradation-prone surfaces. Mechanistically, this mode of stabilization serves as a primary mechanism for driving tumor immune evasion; for instance, L-lactylation shields the immune checkpoint protein PD-L1 from ubiquitin-mediated degradation, thereby stabilizing its expression and promoting an immunosuppressive environment [87]. Similar stabilizing effects predominate across other documented substrates, including BLM, PTBP1, and AKR1B10, where L-lactylation suppresses the ubiquitination of these proteins and extends their half-life [18, 53, 68, 88]. Nevertheless, this modification is not uniformly protective. L-lactylation of Axin1 and YTHDC1 promotes their ubiquitin-mediated degradation [60, 89], indicating that this modification can selectively preserve or eliminate specific protein pools depending on the molecular context. Furthermore, L-lactylation also affects nonproteasomal degradation pathways, influencing autophagy and lysosomal targeting through substrates such as FLCN and ENO1 [55, 90]. These data suggest that L-lactylation functions as a metabolically responsive proteostasis switch, enabling cells to selectively remodel the proteome during high glycolytic flux or therapeutic-induced stress.

### Regulation of subcellular localization

Lysine L-lactylation modulates protein subcellular localization by altering surface charges or facilitating the conformational unmasking of cryptic localization signals (Fig. 3d). It promotes nuclear translocation by inducing conformational changes that promote interactions with nuclear import machinery. For instance, L-lactylation of XRCC1 at K247 strengthens its affinity for nuclear transport receptors, subsequently increasing DNA repair efficiency [82]. Similarly, L-lactylation of ABCF1 at K430 exposes the C-terminal nuclear localization signal, facilitating its nuclear entry and indirectly upregulating KDM3A expression [36]. Additionally, cytoplasmic RIG-I relocalizes to the nucleus upon L-lactylation, where it competitively inhibits PARP1 auto-PARylation, demonstrating functional conversion driven by spatial redistribution [91]. Conversely, L-lactylation induces nuclear retention by obstructing export pathways: YAP K90 L-lactylation sterically disrupts CRM1 binding, resulting in its nuclear accumulation and the sustained transcription of oncogenic targets [92]. Furthermore, L-lactylation can inhibit nuclear import to enforce cytoplasmic sequestration. L-lactylation of STAT1 at K193 impairs its interaction with JAK2, preventing STAT1 phosphorylation, dimerization, and subsequent nuclear translocation, which effectively inactivates interferon signaling [93]. In addition to altering its stability, PD-L1 L-lactylation also contributes to its cytoplasmic retention [62]. Collectively, these examples establish that L-lactylation-driven spatial redistribution serves as a critical regulatory layer, often cooperating with PPI modulation or transcriptional regulation to dictate downstream cellular functions.

### Modulation of nucleic acid binding affinity

Lysine L-lactylation directly affects genome and transcriptome dynamics by altering protein‒nucleic acid interactions through charge redistribution and steric perturbation (Fig. 3e). L-lactylation of DNA-binding proteins can increase binding affinity by modifying the potential of the interaction surface. For example, multisite HAT1-mediated L-lactylation of RPA1 increases its binding to single-stranded DNA [48], and CBP-catalyzed L-lactylation of MRE11 at K673 increases its DNA binding capacity [44]. L-lactylation similarly dictates RNA recognition by RNA-binding proteins (RBPs). EP300-mediated K92 L-lactylation of the m⁵C reader YBX1 enhances its interaction with m⁵C-modified transcripts to promote oncogene expression [70]. Furthermore, EP300-driven L-lactylation of METTL3 increases its binding to target RNAs, thereby increasing the m⁶A methylation efficiency of downstream transcripts [41]. These findings highlight L-lactylation as a fundamental regulator of nucleic acid‒protein interactions, dynamically coupling metabolic states to both transcriptional and epitranscriptomic programming.

### Emerging role in regulating liquid‒liquid phase separation

Lysine L-lactylation may influence liquid–liquid phase separation (LLPS) by modulating the electrostatic and hydrophobic properties of lysine residues within intrinsically disordered regions (IDRs), where multivalent, low-affinity interactions drive condensate formation [94–96]. Biochemically, the β-hydroxyl group of the L-lactyl moiety imparts this molecule with amphiphilic characteristics, which expands the local hydrophobic interface while maintaining hydrogen bonding capacity, thereby fundamentally recalibrating the interaction networks within IDRs [96]. Functionally, L-lactylation can either promote or inhibit LLPS. Under hypoxic conditions, EP300-catalyzed K82 L-lactylation of the nuclear m⁶A reader YTHDC1 increases condensate formation, sequestering and protecting target mRNAs such as HIF1A to promote the progression of clear cell renal cell carcinoma [45]. Conversely, AARS1-mediated L-lactylation of lysines within the DNA-binding domain of p53 impairs its ability to undergo LLPS and disrupts its transcriptional activation hubs, leading to the downregulation of tumor suppressor genes and tumorigenesis [57]. Whether L-lactylation universally dictates phase separation or operates in a strictly protein-specific context remains unclear. In vitro reconstitution experiments utilizing site-specific L-lactylated proteins are needed to directly evaluate the intrinsic phase separation properties conferred by this modification.

## Functional adaptation landscape in cancer

Metabolic reprogramming is a fundamental hallmark of cancer and typically results in excessive lactate accumulation [24]. Rather than acting merely as a passive metabolic byproduct, the accumulated L-lactate serves as a primary acyl donor for the L-lactylation of critical metabolic enzymes and signaling regulators. Consequently, non-histone L-lactylation functions as a critical metabolic switch that regulates tumor physiology. It integrates oncogenic signaling, DNA repair, cell death evasion, stemness maintenance, and tumor microenvironment (TME) adaptation. Through these pathways, L-lactylation translates metabolic fluctuations into adaptive cellular programs that accelerate tumor progression. Although the substrates surveyed below are numerous, they are governed by a limited set of recurring molecular operations already defined in Section "Molecular basis of functional regulation: How L-lactylation alters protein function": (i) remodeling of protein–protein and protein–nucleic-acid interfaces to license or disband complexes; (ii) allosteric or kinetic modulation of enzyme activity and oligomeric state; (iii) evasion of ubiquitin–proteasomal degradation to stabilize oncogenic effectors; and (iv) redistribution of proteins between compartments or phase-separated condensates. Each cancer hallmark that follows could be read as the redeployment of these same operations toward a different functional output. Accordingly, we will name the dominant operation for each subsection.

### Metabolic reprogramming: Self-reinforcing circuitry and metabolic flexibility

#### Self-reinforcing feedback loops in metabolic reprogramming

At the enzymatic level, L-lactylation of ENO1 significantly increases its binding to PKM1 and PKM2, thereby facilitating glycolytic complex assembly [19, 90]. Furthermore, L-lactylation can promote lactate production by enhancing the enzymatic activity of non-histone proteins. For example, K115 L-lactylation of PKM2 drives EMT in the context of clear cell renal cell carcinoma [97]. Similarly, PFKM K678 L-lactylation increases glucose uptake and lactate production in Ishikawa cells [98]. Moreover L-lactylation of LDHA at K81 and K318 increases its enzymatic activity, forming a positive feedback loop that elevates the overall cellular lactylation level [52]. Beyond direct modulation of catalytic activity, targeted evasion of protein degradation constitutes a secondary regulatory layer. For instance, PSMD14 L-lactylation increases its deubiquitinase activity to indirectly stabilize ENO1 [99]. Furthermore, the direct L-lactylation of SHMT2, HK2, AKR1B10 (K173), ALYREF (K171), and PFKP (K392) protects these proteins from ubiquitin-mediated degradation. This stabilization sustains glycolysis, lactate flux [76, 100, 101] and therapeutic resistance [53, 102]. Notably, L-lactylation of Axin1 indirectly accelerates its degradation, thereby removing a negative checkpoint on glycolysis [89]. However, the finding relies on correlative observations in a single cell model, and their broader relevance remains to be validated. Additional mechanisms, including HNRNPA1-mediated PKM2 alternative splicing and the L-lactylation of signaling adaptors (such as ABCF1, IGF1R, NUSAP1, and NSUN2), effectively integrate metabolic reprogramming with transcriptional control and oncogenic amplification [36–38, 103, 104].Across these examples the dominant operation is degradation-evasion and allosteric enzyme control (operations i–iii), redeployed to reinforce glycolytic flux. Importantly, this system retains flexibility because negative feedback pathways can eventually counterbalance excessive flux to preserve metabolic homeostasis and dynamic adaptability.

#### Dynamic regulation of metabolic flexibility

Beyond establishing steady-state positive feedback, non-histone L-lactylation provides cancer cells with profound metabolic plasticity, permitting rapid transitions between nutrient utilization pathways in response to fluctuating microenvironmental stress. For example, KAT8-mediated L-lactylation of MDH2 at K239 increases its catalytic efficiency, increases the NADH/NAD⁺ ratio and mitochondrial ATP production, and accelerates citrate export via MDH2-CS-SLC25A1 complex assembly [71, 105, 106]. In the pentose phosphate pathway (PPP), K45 L-lactylation of glucose-6-phosphate dehydrogenase (G6PD) drives the dimerization of this enzyme and NADPH production in a highly context-dependent manner [107–109]. Moreover, L-lactylation of DCBLD1 at K172 stabilizes this protein to suppress the autophagic degradation of G6PD, further sustaining PPP flux [110]. These cases collectively indicate that L-lactylation strongly influences both canonical glycolysis and parallel branches of carbon metabolism.

Beyond this, L-lactylation-mediated regulation extends to diverse secondary metabolic networks. EP300-catalyzed NMNAT1 K128 L-lactylation stabilizes nuclear NAD⁺ salvage to reverse NAD⁺ depletion [46]. In the context of hepatocellular carcinoma, PD-L1 K189 L-lactylation suppresses cholesterol biosynthesis [62], whereas LDHC4-induced ACAA2 K214 L-lactylation increases its catalytic activity, accelerating fatty acid β-oxidation and free fatty acid accumulation to induce autophagy and promote TNBC progression in vitro and in vivo [111]. IGF2BP3 K76 L-lactylation upregulates PCK2 to reprogram serine metabolism, increasing S-adenosylmethionine levels and promoting Lenvatinib resistance in hepatocellular carcinoma [112]. Collectively, these examples establish L-lactylation as a context-dependent regulatory switch with remarkable specificity that enables cancer cells to dynamically sense metabolic cues and continuously alter enzymatic kinetics, redox balance, and metabolic flux across diverse biochemical pathways.

#### Negative feedback regulation and metabolic complexity

**Fig. 4:**
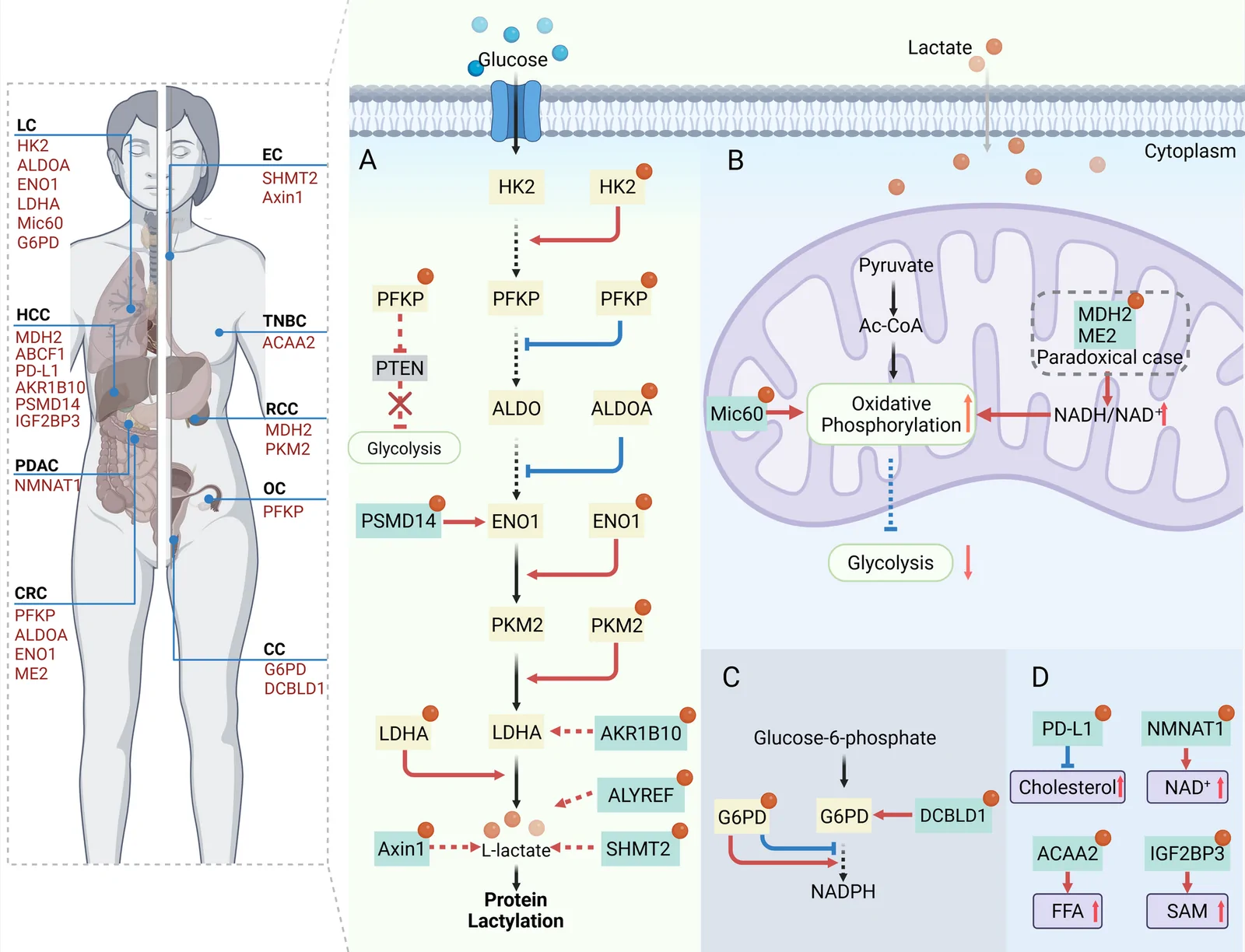
L-lactylation-mediated metabolic rewiring and flexibility in cancer. **A** Glycolysis: L-lactylation of enzymes including HK2, PFKP, ALDOA, PSMD14, ENO1, PKM2, LDHA, AKR1B10, ALYREF, Axin1, and SHMT2 directly modulates glycolytic flux. **B** Mitochondrial oxidative phosphorylation: Modifications of Mic60, MDH2, and ME2 indirectly regulate the balance between glycolysis and respiration. Lactylation represses oxidative phosphorylation in most cases, yet lactylation of Mic60 and MDH2 exerts a paradoxical promoting effect on this process. **C** Pentose phosphate pathway (PPP): G6PD and DCBLD1 L-lactylation redirects metabolic flux toward the PPP. **D** Alternative metabolic branches: PD-L1 L-lactylation alters cholesterol metabolism; NMNAT1 L-lactylation promotes NAD⁺ salvage; ACAA2 L-lactylation accelerates free fatty acid (FFA) synthesis; and IGF2BP3 L-lactylation enhances serine metabolism to increase S-adenosylmethionine (SAM) production. LC, Lung Cancer; HCC, Hepatocellular Carcinoma; PDAC, Pancreatic Ductal Adenocarcinoma; CRC, Colorectal Cancer; EC, Esophageal Cancer; TNBC, Triple-Negative Breast Cancer; RCC, Renal Cell Carcinoma; OC, Ovarian Cancer

Negative feedback mechanisms of L-lactylation exist to prevent metabolic overdrive. In the context of lung adenocarcinoma, L-lactylation of the mitochondrial cristae protein Mic60 paradoxically increases oxidative phosphorylation [113], and ME2 K352 L-lactylation promotes malate-to-pyruvate conversion to maintain mitochondrial respiration and redox homeostasis indicating that this modification does not uniformly favor glycolysis [72]. ALDOA K147 L-lactylation nearly inhibits the activity of this enzyme, functioning as a metabolic safety valve against fatal cellular acidosis [7, 33]; K688 L-lactylation of PFKP directly suppresses PFKP activity [7]. The evidence for L‑lactylation’s inhibitory effect on glycolysis is limited, and its physiological relevance and in vivo generality await systematic validation. These exceptions confirm that non-histone L-lactylation is characterized by high substrate and site specificity. Its functional direction is tightly coupled by the localized protein structural context and the surrounding cellular environment, precluding simplistic unidirectional generalizations (Fig. 4).

An important open question is whether the lactate transport machinery underlying this metabolic circuitry is itself a lactylation substrate. Despite extensive lactylome mapping in hepatocellular carcinoma, gastric, and prostate cancer models, no study to date has reported lactylation of the monocarboxylate transporters MCT1/MCT4 or of glucose transporters (GLUTs), even though both are central to the glycolytic flux that drives lactate production. The closest available evidence involves LDHB, the enzyme that reconverts imported lactate to pyruvate: de-lactylation of LDHB at K58 relieves STAT1-mediated repression of SLC7A11 and promotes ferroptosis resistance (Section "Resistance to cell death: Beyond apoptosis") [114]. This modification, however, was characterized within a single lactate-exposed tumor cell population, and therefore reflects an intracellular feedback response rather than the classical inter-cellular lactate shuttle, in which lactate secreted by glycolytic donor cells is taken up and reoxidized by neighboring recipient cells. Whether MCT1/4, GLUTs, or shuttle-engaged LDHB in recipient cells are lactylated during this donor–recipient exchange remains untested and represents a priority for future lactylome profiling of the tumor microenvironment.

### Oncogenic signaling and phenotypic plasticity

Non-histone lysine L-lactylation drives oncogenic signaling, cancer stem cell (CSC) maintenance, and cytoskeletal dynamics by stabilizing critical signaling kinases, transcription factors, RNA-binding proteins, and structural effectors.

#### Direct modification of kinase and GTPase activation

L-lactylation of core signaling nodes occurs at critical functional regions. L-lactylation exerts its effects mainly through two mechanisms: allosteric activation of catalytic activity and inhibition of ubiquitin-dependent degradation. In the first mechanism, L-lactylation directly enhances kinase function: K415 L-lactylation of PIK3CB activates this kinase while inhibiting the ULK1-mediated phosphorylation of PSMA4, reinforcing cGAS-mediated tumor-promoting signals [115]. NME1 K49 L-lactylation enhances nucleotide supply and alleviates replication stress [116], but this awaits systematic validation. In the second mechanism, L-lactylation competes with ubiquitination on adjacent or the same lysine residues: CCNE2 L-lactylation stabilizes the protein and promotes the G1/S transition of the cell cycle, thereby increasing tumor proliferation, migration, and invasion [117]. RHOA L-lactylation stabilizes its GTP-bound active state and activates the downstream ROCK/MLC pathway to drive cell proliferation and invasion [118]. In hepatocellular carcinoma, RET K549 L-lactylation similarly blocks ubiquitination to hyperactivate MAPK/PI3K-AKT signaling [119]. The L-lactylation of CEACAM6, TPX2, and SLC26A3 stabilizes these effectors to sustain survival pathways [120–122].

Notably, these modifications frequently establish self-reinforcing loops: Lactate-induced GCN5-mediated ERK L-lactylation increases kinase activity, which reciprocally increases the phosphorylation of GCN5 to amplify proliferative signaling [123]. A preliminary study has suggested that L-lactylation of IGF1R may enhance its ligand affinity, thereby potentially upregulating glycolytic gene expression [103]. Unlike linear signaling cascades, these L-lactylation-driven loops exhibit profound signal amplification and robustness, conferring continuous oncogenic output even in the absence of an initial metabolic stimulus.

#### Transcription factors and chromatin regulatory proteins

In the nucleus, non-histone lysine L-lactylation directly affects DNA binding affinity, nuclear localization, and transcriptional activation, broadly participating in transcriptional regulation through multiple modes. One such mode involves the stabilization of transcriptional activators: NUSAP1 L-lactylation has been reported to stabilize the protein and promote glycolytic gene expression [37], while SOX9 lactylation is also observed to enhance its stability and upregulate EMT-associated genes [124]. However, these studies still require further verification. Another mechanism is enhancing the DNA binding ability of transcription factors or chromatin regulators: K183 L-lactylation of YY1 enhances the binding of this protein to the FBXO33 promoter, which subsequently promotes p53 ubiquitination and degradation to facilitate EMT [125]. CENPA K124 and ZMIZ1 L-lactylation enhance their chromatin association to upregulate oncogenic and stemness factors, including Nanog [126–128]. Furthermore, L-lactylation also influences transcriptional output by regulating nuclear translocation and retention: L-lactylation enables the nuclear translocation and transcriptional activation of Snail1, TWIST1, and ABCF1 (K430), driving EMT and proliferation [36, 129, 130]. YAP K90/K102 L-lactylation promotes YAP nuclear retention, substantially increasing endometrial cancer stemness and tumorigenic potential [92, 131]. In addition, SP1 K19 L-lactylation activates CENPL transcription, which is required for maintenance of the cancer stem cell phenotype [132]. A more detailed summary of how L-lactylation maintains the cancer stem cell phenotype is provided at the end of Section "Oncogenic signaling and phenotypic plasticity". Additional modifications, including ATRX K584 and NFIL3 K360 L-lactylation, regulate specific chromatin interactions and transcriptional repression [10]; L-lactylation of HDAC1 K412 decreases H3K27ac levels at the FTO and ALKBH5 promoters, and this, in cooperation with EPAS1/CEBPD, drives EMT, exacerbating tumor progression [65, 66].

#### Bipartite transcriptional control via L-lactylation dynamics

A few studies have suggested that lactoylation may amplify transcriptional output by forming positive feedback loops with other PTMs or transcription factors. NOL6 K54 L-lactoylation has been reported to promote the YY1/c-Myc loop [133], but its full validation still requires further supplementation. Conversely, the targeted de-L-lactylation of specific factors can also execute oncogenic programs. For instance, the de-L-lactylation of PD-L1 paradoxically increases the YY1-mediated transcription of SQLE, driving aberrant cholesterol biosynthesis and promoting malignancy [62]. Furthermore, L-lactylation can act as a direct transcriptional repressor. AARS1-mediated L-lactylation of p53 at K120/K139 abolishes its DNA binding capacity and disrupts its ability to undergo liquid‒liquid phase separation (LLPS) [57]. This effectively suppresses the expression of critical tumor suppressor genes (such as p21 and PUMA), thereby promoting tumor progression [57].

These examples highlight that the dynamic, reversible interplay between L-lactylation and de-L-lactylation modulates tunes diverse transcriptional outputs, integrating the cellular metabolic state with complex gene regulatory networks.

#### Translation machinery and RNA-binding proteins

Non-histone lysine L-lactylation modulates protein synthesis by regulating RNA-binding proteins (RBPs), ribosome biogenesis, and translation elongation. This leads to the formation of a direct biochemical bridge between metabolic cues and downstream protein production. L-lactylation alters the conformation and RNA binding affinity and regulates the phase separation of RBPs, thereby controlling alternative splicing, RNA modifications (m6A and m5C), and overall mRNA stability.

L-lactylation significantly increases the recognition and stability of specific mRNAs. RBM15 K850 L-lactylation promotes robust binding to the METTL3-WTAP complex. This increases global m6A levels and upregulates target gene expression [64]. YTHDC1 K82 L-lactylation drives the formation of nuclear condensates. This phase separation protects oncogenic mRNAs, such as BCL2 and E2F2, from degradation, substantially extending their half-lives and promoting renal cell carcinoma cell proliferation, migration, and invasion and rapid tumor growth [45, 134]. Similarly, RCC2 K124 L-lactylation recruits SERBP1 to stabilize MAD2L1 mRNA, supporting aberrant cell division [50], whereas NCL K477 L-lactylation stabilizes MADD mRNA by preventing nonsense-mediated decay. This facilitates MAPK/ERK activation and promotes iCCA progression [40]. L-Lactylated YBX1 interacts with G3BP2 to stabilize m5C-modified oncogenic transcripts, promoting cellular proliferation and invasion [70]. Moreover, K692/ K356 L-lactylation of NSUN2 stabilizes key target transcripts and reinforcing oncogenic metabolic feedback loops [27, 104, 135]. Conversely, in specific contexts, L-lactylation can reduce mRNA stability. L-lactylation of YTHDC1 can promote its own ubiquitination and degradation, downregulate JUND expression and accelerate bladder cancer growth [60].

At the level of alternative splicing regulation, lactylation also primarily exerts a promoting effect. HNRNPC K176 L-lactylation increases its tetramer formation and drives exon skipping in PAK6, resulting in the production of highly oncogenic isoforms [136]. The L-lactylation of HNRNPA1 directs PKM pre-mRNA splicing toward the oncogenic PKM2 isoform [38]. And L-lactylation stabilizes the hnRNPA1 protein, directly promoting the generation of the AR-V7 variant in prostate cancer [39]. Furthermore, it has been reported that L-lactoylation at the K43 site can stabilizes the ESRP1 protein, promotes the formation of circPHGDH, thereby driving the malignant progression of tumors [137]. However, more independent studies are still needed for repeated verification. Conversely, L-lactylation can promote mRNA degradation. YTHDF3-mediated suppression of KDM6B compromises DNA damage responses and promotes chemoresistance [9, 138]. However, the L-lactylation of DHX15 at K17 can exert tumor-suppressive effects by stabilizing mature transcripts and inhibiting bladder cancer progression [61].

Beyond RBPs, L-lactylation directly modulates the translation machinery. eEF1A2 K408 L-lactylation increases its catalytic efficiency under aminoacyl-tRNA stimulation, significantly accelerating peptide elongation [85]. Furthermore, eEF2 L-lactylation has been observed in association with enhanced translation fidelity [139]. Collectively, these modifications link metabolic reprogramming directly to the protein synthesis apparatus, enabling cancer cells to sustain the production of very high levels of pro-proliferative proteins even under severe nutrient stress.

#### Tumor cell stemness maintenance

Non-histone lysine L-lactylation functions as a central metabolic switch in maintaining cancer stem cell (CSC) self-renewal and tumorigenic potential. It serves to integrate external signaling pathways, transcriptional programs, and metabolic cues to reinforce cell identity and proliferative capacity.

##### Coregulators of stemness-related transcription factors 

L-lactylation increases the activity and stability of stemness-associated transcription factors. In terms of enhancing activity, SP1 L-lactylation promotes transcriptional programs that maintain the stem cell phenotype, driving proliferation, invasion, and sphere formation [132]. In terms of enhancing stability, L-lactylation prevents the ubiquitination and increases the stability of ANTXR1 and activates RhoC/ROCK1–SMAD5 signaling to enforce the transcription of stemness-related genes [140]. SOX9 L-lactylation upregulates the expression of stemness markers (Oct4, Sox2, and Nanog) and EMT-related genes, thereby reinforcing CSC identity [124]. Notably, L-lactylation of PKM2 does not enhance its enzymatic activity but rather facilitates its interaction with FOSL1, forming superenhancers that activate SNAI2, MYC, and MAZ, thereby sustaining stemness [141].

##### RNA splicing and translation regulators

At this level, L-lactylation also exerts multilevel effects. The L-lactylation of c-Myc increases protein stability and expression, promoting the hypoxia-induced proliferation, invasiveness and stemness and glycolytic reprogramming in HCC cells [142]. PTBP1 K436 L-lactylation stabilizes PFKFB4 transcripts by blocking UPF1-mediated nonsense-mediated decay, establishing a glycolysis–lactate–L-lactylation feedback loop that reinforces stemness in glioma stem cells [68]. Lactate-induced L-lactylation of SPTAN1 increases its interaction with NOTCH1, promoting HES1 transcription and CSC maintenance [143]. Conversely, the L-lactylation of SLC26A3 at K536 reduces the stability of this protein, indirectly modulating the HuR/CUGBP1-mediated mRNA regulation of stemness markers (CD44, CD133, and E-cadherin), highlighting the bidirectional control effects of this modification [122]. Taken together, L-lactylation cooperatively maintains CSC properties through three routes: stabilizing proteins, protecting key metabolic transcripts, and reinforcing pro-stemness signaling interactions, with the specific effects governed by the modification sites and downstream effectors.

##### Chromatin remodeling

L-lactylation plays a crucial role in telomere maintenance and chromatin architecture, which are essential for maintaining CSC identity. L-lactylation of PARP1 at multiple sites upregulates adhesion complexes and CTCF while downregulating TERF2. This contributes to telomere length and promotes liver CSC stemness [144]. Conversely, POLR2A L-lactylation can suppress specific stemness pathways by inducing transcriptional pausing, inhibiting R-loop clearance, and attenuating Wnt signaling [145].

These findings underscore that L-lactylation orchestrates CSC maintenance through the coordinated regulation of transcription factors, RNA processing, and chromatin remodeling while also allowing the context-dependent suppression of stemness, illustrating its versatile and bidirectional role in tumor cell identity.

#### Cytoskeletal remodeling and cell motility

Non-histone lysine L-lactylation directly modulates cytoskeletal regulators and structural proteins, increasing tumor cell migration, invasion, and metastatic potential by altering filament dynamics and cellular mechanics. CFL1 K22 L-lactylation prolongs the half-life of cofilin 1, leading to actin cytoskeleton remodeling, reduced cell stiffness, and diminished adhesion, thereby driving the malignant progression of nasopharyngeal carcinoma [54]. PRMT1 L-lactylation increases its methyltransferase activity, promoting the catalysis of vimentin asymmetric dimethylation, which stabilizes intermediate filaments, promotes EMT, and facilitates tumor cell motility and distant metastasis [146].

Importantly, L-lactylation does not universally promote malignancy. Lactylation of PPP1R14B at K140 suppresses the proliferation and migration of cervical cancer cells [147]. Loss of this modification increases malignancy, indicating that L-lactylation can also act as a regulatory brake on cytoskeletal dynamics and cell cycle progression in specific molecular contexts.

### Genomic stability and the DNA damage response

Non-histone lysine L-lactylation exerts plays a key role in DNA damage repair (DDR) mechanisms by regulating pathways such as homologous recombination (HR), nonhomologous end joining (NHEJ), base excision repair (BER), and single-strand break repair (SSBR). During these processes, L-lactylation exerts context‑dependent regulatory effects at distinct repair stages by targeting multiple core repair factors. Such modulation is achieved through alterations in protein conformation, protein‒DNA interactions, and complex assembly.

#### Regulation of homologous recombination (HR)

HR depends on the precise timing and coordinated assembly of repair complexes [148]. In the damage-sensing stage, The L-lactylation of NBS1 at K388 by TIP60 promotes conformational changes that stabilize the MRE11–RAD50–NBS1 (MRN) complex. This promotes the recruitment of downstream repair proteins and is correlated with chemotherapy resistance [17, 149, 150]. In the end-resection stage, CBP-mediated MRE11 K673 L-lactylation strengthens DNA-end binding, facilitating 5′-end resections [44, 151]. RPA1 L-lactylation increases ssDNA coating and scaffolding for HR factors such as BLM and MRE11 [47, 48]. BLM K24 L-lactylation maintains protein stability, supporting DNA2-mediated end processing [18]. Furthermore, upon progression to the strand-invasion stage, RAD51 L-lactylation promotes nucleoprotein filament formation and accelerates recombination [152]. Collectively, these modifications modulate HR efficiency and continuity by reinforcing stable interactions at each stage of repair.

#### Regulation of NHEJ and other DNA damage repair pathways

L-lactylation of factors in the NHEJ pathway primarily accelerate break-end closure by enhancing protein‒DNA and protein‒protein interactions and increasing the efficiency of complex assembly. During the damage-sensing and end-binding phases, L‑lactylation modifications have been identified at multiple lysine residues of Ku70 (K237, K287, K461, K539, K570, K591, K596) and Ku80 (K543), which are thought to potentially enhance heterodimer stability and DNA‑PKcs recruitment [11, 153]. Moreover, cooperativity between lactylated Ku scaffold protein and FEN1 is also speculated to promote the recruitment of end‑processing factors [11, 52, 153]. At the stages of complex stabilization and end-bridging, GCN5-mediated L-lactylation of XLF at K288 expands the interaction surface with Ku70/80, promoting XRCC4-LIG4 complex assembly and stabilizing DNA-end bridging [49, 154, 155]. In other DNA damage repair pathways, the L-lactylation of XRCC1 at K247 increases its nuclear translocation for base excision repair [82]. Furthermore, L‑lactylation of PARP1 at K654 has been reported to enhance poly(ADP‑ribosyl)ation activity and may be associated with accelerated nucleotide excision repair [69, 156].

#### Context-dependent inhibition of the DDR

L-lactylation does not universally promote the DDR. RIG-I L-lactylation sequesters PARP1, severely attenuating HR capacity [91]. At the level of key regulatory factor sequestration and signaling interference, L-lactylation of ARHGDIB at K47/K50 leads to Rac1 sequestration, suppressing the activity of the Rac1-ATM-MRN-CHK2 axis and reducing HR and NHEJ efficiency [63]. In terms of epitranscriptomic regulation, METTL3 L-lactylation at K27 reduces m6A levels on DDR-related transcripts, sensitizing triple-negative breast cancer cells to cisplatin [42]. AARS2-mediated YTHDF3 lactylation stabilizes this protein and promotes the degradation of KDM6B mRNA, impairing DDR initiation [9]. This mechanism can suppress bladder cancer progression and increase chemosensitivity.

**Fig. 5:**
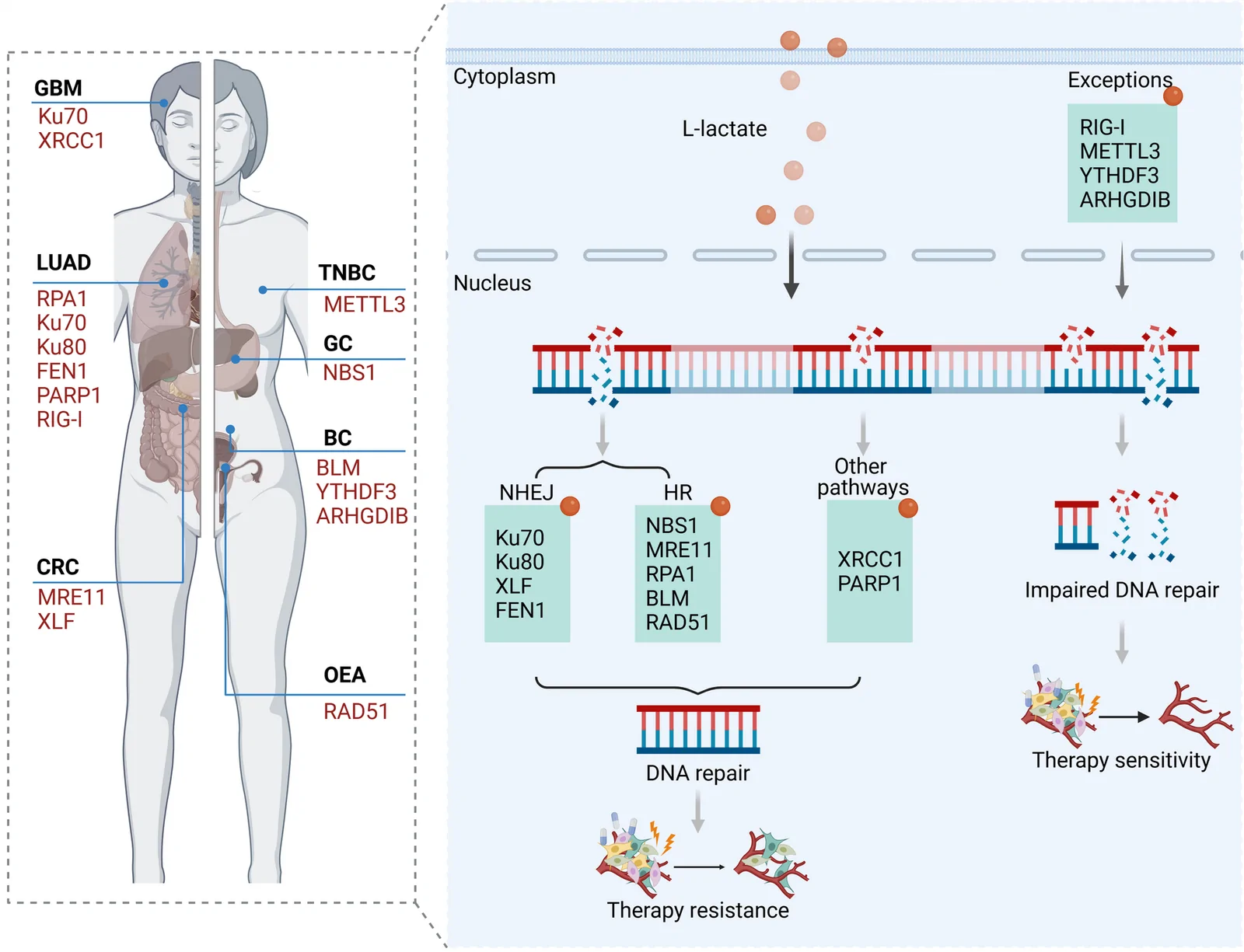
L-lactylation networks governing the DNA damage repair (DDR). Nuclear L-lactylation modifies key DDR factors across multiple repair cascades, including homologous recombination (HR; NBS1, MRE11, RPA1, BLM, RAD51), non-homologous end joining (NHEJ; Ku70, Ku80, XLF, FEN1), and Other DNA repair pathways (XRCC1, PARP1). L-lactylation generally enhances DNA repair capacity and preserves genomic stability under genotoxic stress, thereby mediating therapeutic resistance. Conversely, specific L-lactylation events (e.g., on RIG-I, METTL3, YTHDF3, and ARHGDIB) can impair DNA repair functions and sensitize tumor cells to therapy. GBM, Glioblastoma; LUAD, Lung Adenocarcinoma; CRC, Colorectal Cancer, TNBC, Triple-Negative Breast Cancer; GC, Gastric Cancer; BC, Bladder Cancer; OEA, Ovarian Endometrioid Adenocarcinoma

These examples demonstrate that the impact of L-lactylation on DNA repair depends on the specific protein targets and modification sites. However, most reports indicate that non-histone L-lactylation tends to increase DNA repair capacity. This systematic bias has clear clinical significance for therapeutic resistance and is discussed further in Section "Therapeutic resistance: L-lactylation as a convergent adaptive hub" (Fig. 5).

### Resistance to cell death: Beyond apoptosis

Non-histone lysine L-lactylation confers exceptional survival advantages to tumor cells, primarily through the extensive regulation of nonapoptotic cell death pathways. This includes the modulation of ferroptosis, pyroptosis, cuproptosis, and autophagic survival programs while also governing classical apoptosis. This highlights the multifaceted and critical role of lysine L-lactylation in driving profound resistance to cell death.

#### Ferroptosis resistance

Non-histone lysine L-lactylation confers ferroptosis resistance through both the direct stabilization of key executioners and antioxidant enzymes and indirect epigenetic and posttranscriptional regulation. Regarding the former, GPX4 L-lactylation prolongs the half-life of this protein [157]. L-lactylation of PCBP2 at K115 inhibits its ubiquitination and reduces iron-induced oxidative stress [158]. Furthermore, L-lactylation of PSMD14 at K100 stabilizes ENO1, which further increases lactate production [99]. PRDX1 L-lactylation augments ROS scavenging and activates NRF2 [159]. GCLM K34 L-lactylation increases glutamate–cysteine ligase activity to increase glutathione (GSH) synthesis and limit lipid peroxidation [160]. These direct mechanisms construct a defense barrier against ferroptosis in tumor cells at the levels of enzyme activity and stability, substrate supply, and redox balance. Regarding indirect mechanisms, L-lactylation typically regulates ferroptosis-related proteins through transcriptional regulation and protein stability. HDAC1 K412 L-lactylation suppresses FTO and ALKBH5, increasing m6A levels on FSP1 mRNA [65]. IGF2BP3 L-lactylation increases FSP1 levels [112]. NSUN2 K508 L-lactylation stabilizes GCLC transcripts via m5C modification [161]. Modification of SPRING and LSD1 modulates TRIM21-mediated degradation and reduce TFRC expression [88, 162]. Finally, SUMO2 L-lactylation at K11 promotes the proteasomal degradation of ACSL4 [56]. Ferroptosis resistance can also unexpectedly result from the targeted loss or active reversal of L-lactylation. Specific de-L-lactylation of LDHB at K58 promotes STAT1 degradation, effectively relieving the repression of SLC7A11 and substantially increasing GSH synthesis [114]. Conversely, L-lactylation of PRMT5 at K240 leads to the upregulation of ALKBH5, stabilizing SLC7A11 mRNA [163]. Collectively, these studies establish a multifaceted, context-dependent regulatory network centered largely around SLC7A11 that effectively couples direct enzymatic stabilization with complex epigenetic and posttranscriptional circuits to flawlessly coordinate the susceptibility of tumors to ferroptosis.

#### Pyroptosis, cuproptosis, and autophagy

Non-histone lysine L-lactylation orchestrates a network of nonapoptotic cell death pathways, increasing tumor cell survival under metabolic or therapeutic stress. In the context of pyroptosis, L-lactylation of PKM2 at K433 blocks its nuclear translocation and the NF-κB-dependent expression of NLRP1, Caspase-1, GSDMD, and IL-1β. Moreover, CNPY3 K215/K224 L-lactylation preserves lysosomal integrity, preventing cathepsin B release and caspase activation [67, 164, 165]. At present, the evidence in this regard is not sufficient and further research is needed to clarify it.

L-lactylation modulates cuproptosis in a highly bidirectional manner. L-lactylation of NUDT21 at K23 reduces FDX1 protein levels via CFIm-mediated 3′UTR extension, conferring resistance to cuproptosis in esophageal carcinoma cells [59]. Conversely, METTL16 L-lactylation stabilizes FDX1 mRNA through m6A, promoting DLAT lipoylation and cell death [21].

L-lactylation also promotes adaptive survival via autophagy. FLCN L-lactylation inhibits mTORC1 lysosomal localization, facilitating TFEB nuclear translocation and the transcription of autophagy-related genes [55, 166]. Direct L-lactylation of TFEB stabilizes this protein. Furthermore, Vps34 L-lactylation at K356/K781 promotes Beclin1-Atg14L-UVRAG complex formation, increasing lipid kinase activity and accelerating prosurvival autophagic flux [167]. Additionally, modification of ACAA2 and FOXO3 activates prosurvival PI3K/AKT/mTOR signaling, sustaining persistent autophagy and PDAC cell survival [111, 168]. Collectively, these modifications collectively establish lysine L-lactylation as a widespread modulator of nonapoptotic cell death, effectively integrating pyroptosis suppression, cuproptosis modulation, and pro‑survival autophagy to promote treatment resistance.

#### Regulation of apoptosis

Non-histone lysine L-lactylation exerts bidirectional control over apoptosis, modulating tumor cell survival and therapeutic resistance. Suppressive modifications strongly increase cell survival by physically interfering with key apoptotic executioners. In the context of apoptosis inhibition, L‑lactylation primarily constructs an anti‑apoptotic barrier through the synergistic action of three mechanisms: protein–protein interactions, regulation of enzyme activity and translocation (operations i, ii, iv). CASP3 K14 L-lactylation inhibits protease activation, blocking apoptosis in leukemia cells [169]. ACF1 L-lactylation promotes GCLM transcription through increased H3K27ac/H3K4me3 deposition and Myc recruitment, increasing glutathione synthesis and reducing lipid peroxidation [170]. L-lactylation facilitates the mitochondrial translocation of ABHD6, where it competitively inhibits FIS1-mediated DRP1 fission, maintains mitochondrial hyperfusion, suppresses ROS accumulation, and limits mitophagy [171, 172]. Conversely, highly specific tumor-suppressive L-lactylation events can promote apoptosis. One notable example is CA3 K36 L-lactylation, which strongly activates Caspase-3, suppresses DUOX2, markedly lowers intracellular ROS levels and induces terminal apoptosis [173]. Collectively, these studies highlight L-lactylation as a highly context‑dependent modulator of apoptosis that is capable of both promoting survival and enabling cell death, thereby tightly modulating tumor adaptability under extreme stress or direct therapeutic challenge. However, current evidence in this direction remains limited and requires further investigation.

### Regulation of the tumor microenvironment

The tumor microenvironment (TME) is a complex ecosystem composed of tumor cells, infiltrating immune cells, and stromal cell populations [174, 175]. Non-histone L-lactylation serves as a principal metabolic conduit mediating intercellular crosstalk within the TME [176]. This section systematically examines how L-lactylation-driven microenvironment remodeling affects tumor progression, with a specific focus on cytokine networks, immune cell exhaustion, and vascular architecture.

### Targeted evasion of proteasomal degradation in the context of immune checkpoint regulation

In parallel, L-lactylation orchestrates profound immunosuppression not only by altering signaling cascades but also by directly manipulating the proteostatic stability of key immune checkpoints. As recently demonstrated, the L-lactylation of programmed death-ligand 1 (PD-L1) at K271 and K280 acts as a metabolic switch that shields PD-L1 from ubiquitin-mediated proteasomal degradation [87]. By intrinsically stabilizing PD-L1 expression on the tumor cell surface, this specific L-lactylation event strongly suppresses CD8⁺ T-cell cytotoxicity and facilitates robust immune escape [87, 177, 178]. Importantly, this degradation–evasion paradigm reveals a novel therapeutic vulnerability: combinatorial strategies employing dietary interventions (e.g., specific amino acid restriction) alongside immunotherapy can effectively modulate this L-lactylation axis to restore antitumor immunity [87].

Beyond PD-L1 stabilization, L-lactylation broadly reshapes the recruitment and polarization of other immunosuppressive populations. In the context of glioblastoma, L-lactylation of MOESIN at K72 stabilizes the FERM domain, enhances TGF-β receptor engagement, promotes SMAD3 signaling, increases cortical rigidity, and facilitates immunosuppressive synapse formation, increasing Treg activity [179]. Furthermore, SPTAN1 lactylation increases prostaglandin E2 (PGE2) production, promoting exhausted CD8⁺ T-cell infiltration [143, 180]. L-lactylation of the CD44 extracellular domain impairs hyaluronic acid binding and AKT signaling, reducing cytotoxic T-cell function [86].

L-lactylation also drives immunosuppressive cell recruitment and the polarization of immunosuppressive myeloid cells. Rac2 K-GTPase activation via L-lactylation promotes MDSC infiltration and limits CD8⁺ T-cell activity [181]. L-lactylation of APOC2 at K70 prevents its own degradation, leading to the substantial release of free fatty acids that strongly increase Treg accumulation [182]. ENSA L-lactylation at K63 activates SRC/STAT3/CCL2 signaling, recruiting and reprogramming tumor-associated macrophages (TAMs) toward a protumor phenotype [22]. The L-lactylation of EGR1 at K422 and IKZF1 in the nucleus reinforces transcriptional programs that recruit senescence-associated macrophages and compromise the blood–brain barrier [183, 184]. Finally, STAT1 L-lactylation obliterates CD8⁺ T-cell infiltration, promoting immune evasion across multiple aggressive cancer types [93].

### Cytokine dysregulation and immunosuppressive mediators

L-lactylation further modulates the TME by simultaneously suppressing the production of antitumor cytokines and driving the secretion of immunosuppressive mediators. In the context of glioblastoma, Ku70 lactylation inhibits the critical cGAS-STING viral sensing pathway; furthermore, L-lactylation of cGAS at K21/K131 drives its nuclear translocation and degradation, reducing type I interferon production and impairing CD8⁺ T-cell recruitment [115, 185, 186]. Furthermore, STAT1 K193 L-lactylation blocks its interaction with JAK2. This prevents STAT1 phosphorylation, inhibits CXCL9/10/11 chemokine expression, and completely halts cytotoxic T-cell infiltration [93].

Conversely, L-lactylation promotes the expression of immunosuppressive cytokines and checkpoint ligands. METTL3 L-lactylation activates STAT3 to upregulate the expression of IL-6, IL-10, and iNOS, enhancing the immunosuppressive function of TAMs in the context of hepatocellular carcinoma [41]. METTL16 L-lactylation stabilizes CTCF to induce CD276 and NECTIN2 expression in both liver and colorectal cancers [20]. VIRMA L-lactylation increases SP1-mediated TGF-β transcription in cervical cancer [187]. Finally, the L-lactylation of SPTAN1 activates NOTCH1/HES1 signaling, increasing COX2/PGE2 biosynthesis and enhancing immune evasion in hepatocellular carcinoma [143]. Together, these specific modifications effectively couple metabolic cues with cytokine networks to establish a highly immunosuppressive TME.

### Remodeling of the tumor vasculature

**Fig. 6:**
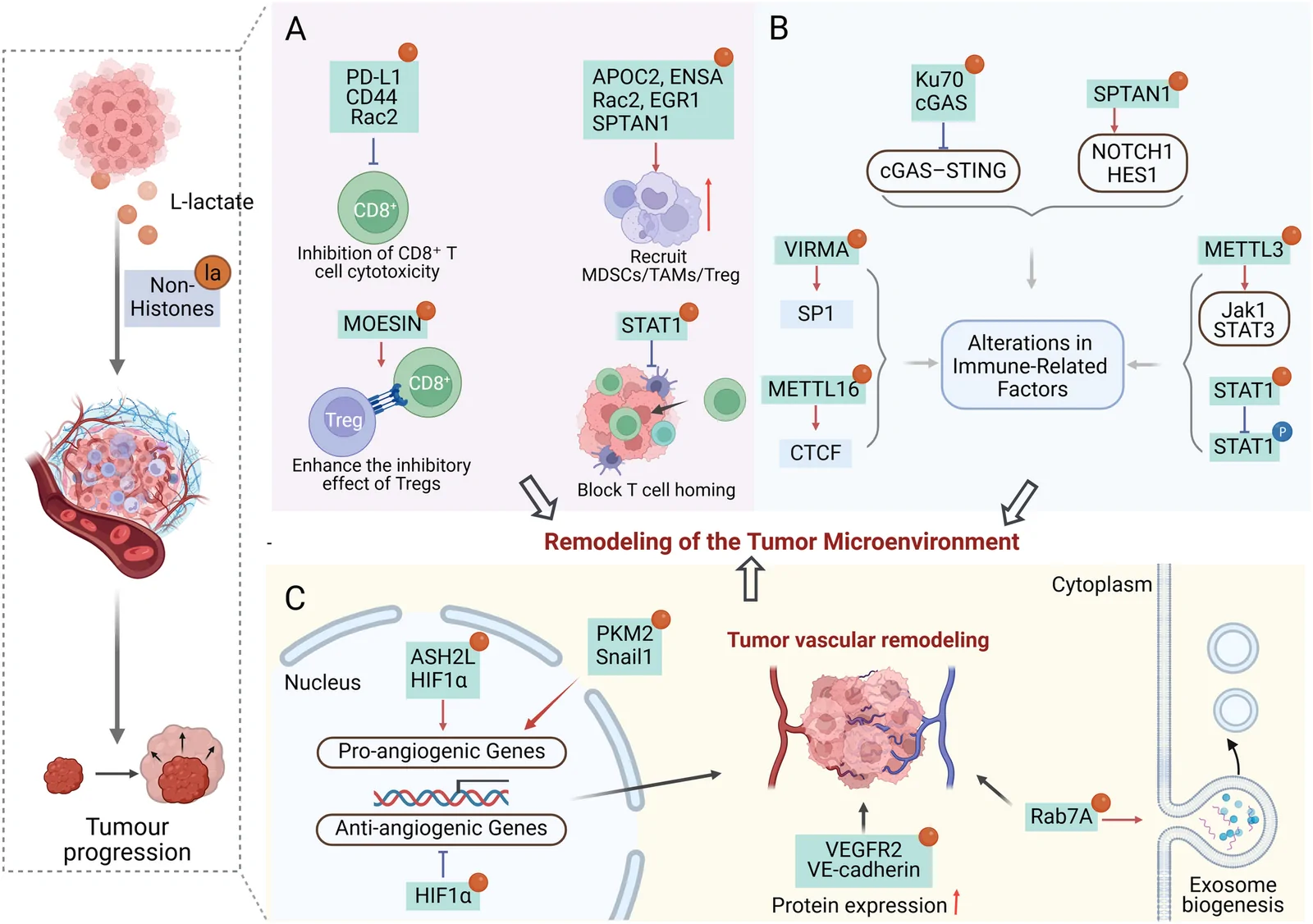
Lactate-driven L-lactylation sculpts an immunosuppressive tumor microenvironment. **A** Immune cell function: Stereospecific L-lactylation of PD-L1 evades proteasomal degradation to constitutively suppress CD8⁺ T cell cytotoxicity. Concurrent L-lactylation of CD44 and Rac2 further impairs T cell activity, while Moesin L-lactylation enhances regulatory T cell (Treg) function. Modifications of APOC2, ENSA, Rac2, EGR1, and SPTAN1 recruit myeloid-derived suppressor cells (MDSCs) and tumor-associated macrophages (TAMs). STAT1 L-lactylation eliminates the infiltration of CD8⁺ T cells. **B** Cytokine networks: L-lactylation of Ku70, cGAS, and SPTAN1 silences the cGAS-STING and NOTCH1-HES1 signaling pathways. Modifications along the STAT1, VIRMA-SP1, METTL16-CTCF, and METTL3-JAK1-STAT3 axes trigger cytokine dysregulation. **C** Vascular remodeling: ASH2L/HIF1α, PKM2, and Snail1 L-lactylation activates angiogenesis and mesenchymal transitions; VEGFR2 and VE-cadherin modifications induce vascular abnormalities. L-lactylation of Rab7A promotes exosome biogenesis, thereby remodeling the vasculature

Non-histone lysine L-lactylation critically regulates tumor angiogenesis, vasculogenic mimicry, and endothelial barrier destruction. These effects facilitate hematogenous dissemination and reshape the physical TME. Existing evidence can be summarized into three main pathways: transcriptional activation, nuclear translocation and signal integration, and vesicle transport and secretion. The L-lactylation of specific epigenetic regulators and transcription factors synergistically activates proangiogenic gene networks, most notably VEGFA. In hepatocellular carcinoma, ASH2L K312 L-lactylation enhances its binding to VEGFA genomic loci and recruits the MLL complex to increase H3K4 methylation, increasing VEGFA expression and secretion [58]. Furthermore, Moreover, L‑lactylation of HIF1α has been found to promote vasculogenic mimicry [188]. At the level of nuclear translocation and signal integration, L-lactylation of PKM2 at K206 (catalyzed by AARS1) drives its nuclear translocation, enabling its interaction with FOSL1 to upregulate stemness-related genes and facilitate vascular mimicry in the context of hepatocellular carcinoma [141]. Snail1 L-lactylation increases transcriptional repression, promoting EMT [129]. Finally, L-lactylation stabilizes endothelial junction and vesicle trafficking proteins. Rab7A L-lactylation increases endosomal binding, late endosome maturation, and exosome secretion [189]. These tumor-derived exosomes, which carry highly prometastatic proteins and RNAs, further remodel and disrupt the surrounding tumor vasculature [189]. The L-lactylation of VEGFR2 and VE-cadherin increases their protein expression, supporting vasculogenic mimicry [190]. Together, these modifications suggest that lysine L-lactylation as an important molecular mediator of tumor vascular remodeling, tightly linking metabolic signals to the transcriptional, structural, and vesicular mechanisms that drive sustained angiogenesis, metastasis, and aggressive tumor progression (Fig. 6).

## Therapeutic resistance: L-lactylation as a convergent adaptive hub

**Fig. 7:**
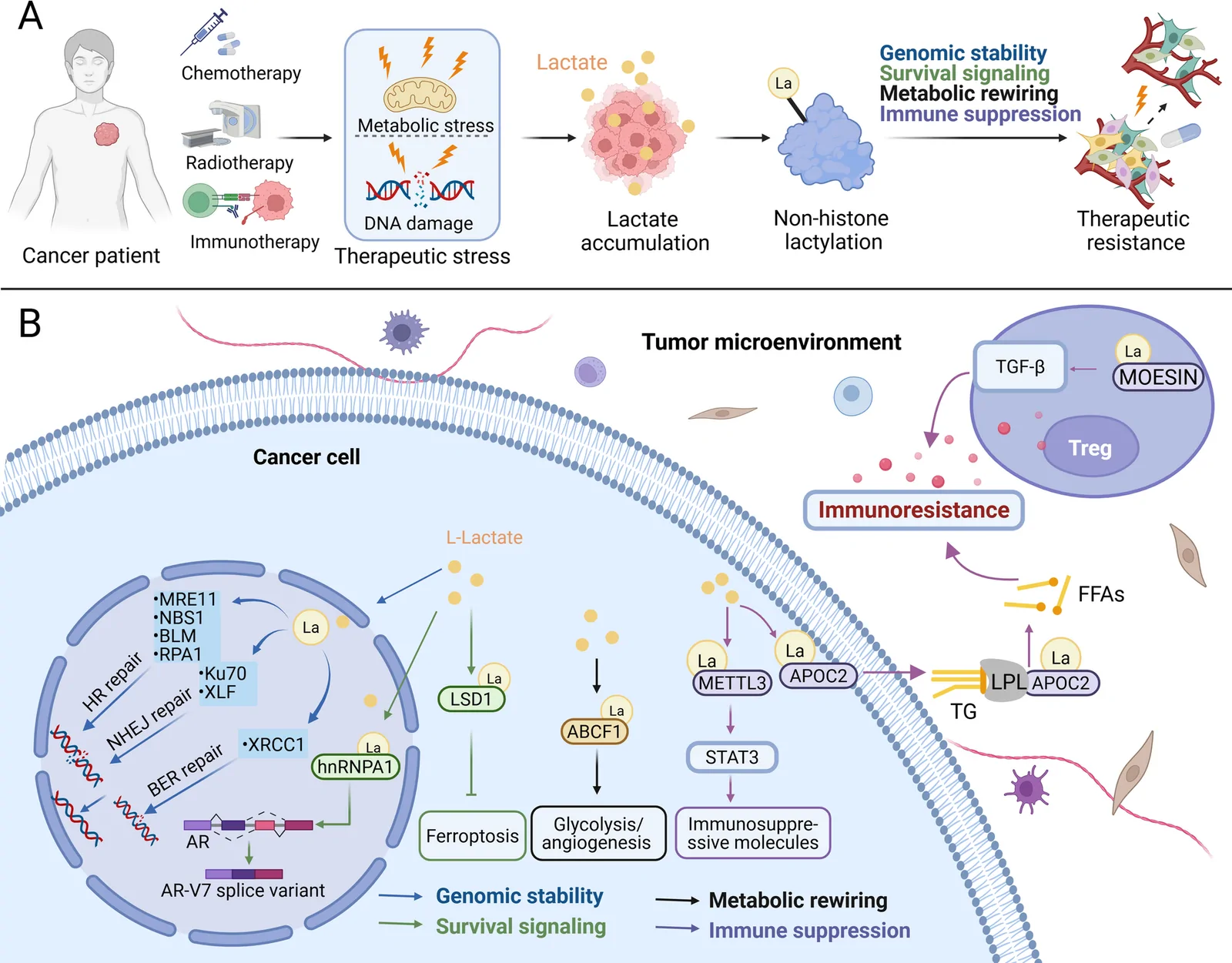
Non-histone L-lactylation as a convergent adaptive node for therapeutic resistance. **A** Anti-cancer interventions (chemotherapy, radiotherapy, and immunotherapy) induce therapeutic stress (metabolic stress and DNA damage), leading to intracellular lactate accumulation and subsequent non-histone L-lactylation, which ultimately drives therapeutic resistance. **B** This metabolically responsive modification acts as a central hub of adaptive resistance via four core axes: maintaining genomic stability by accelerating DNA damage repair to counteract genotoxic stress, activating pro-survival signaling and inhibiting ferroptosis, driving metabolic rewiring, and mediating immunosuppression and immunoresistance in the tumor microenvironment

Therapeutic interventions universally impose extensive stress that exacerbates metabolic reprogramming and intracellular L-lactate accumulation [191]. These microenvironmental stresses are biochemically transduced into adaptive survival mechanisms via non-histone L-lactylation. Specifically, L-lactate-driven modifications dictate resistance through three interconnected mechanisms: (i) accelerating DNA damage repair; (ii) increasing metabolic activity and survival signaling to sustain proliferation; and (iii) abrogating immune surveillance. This biochemical cascade, which progresses from metabolic stress to L-lactylation-mediated functional adaptation, results in a robust phenotype resistant to chemotherapy, radiotherapy, targeted therapy, endocrine therapy, and immunotherapy (Table S2). Consequently, L-lactylation may functions as a convergent node that couples metabolic stress to multimodal therapeutic resistance (Fig. 7). Rather than invoking distinct machinery for each treatment, cancers appear to redeploy a shared, metabolically gated response: therapy-induced stress raises intracellular L-lactate. This, through the non-histone lactylation events described in Sections "Genomic stability and the DNA damage response" "Resistance to cell death: Beyond apoptosis" "Regulation of the tumor microenvironment", is read out as accelerated repair, sustained survival signaling, and immune exclusion. We therefore treat therapeutic resistance not as a new mechanism, but as the convergence of previously described adaptive pathways under a common stress input. The subsections below are organized by treatment modality and cross-reference the underlying events rather than re-deriving them.

### Resistance to genotoxic therapies: Chemotherapy and radiotherapy

Non-histone lysine L-lactylation drives resistance to genotoxic therapies primarily by accelerating DNA repair through both HR and NHEJ mechanisms. MRE11 K673 and NBS1 K388 L-lactylation underlie platinum-drug resistance in gastric, liver, and ovarian cancers [17, 44], while XLF K288 L-lactylation similarly contributes to repair of platinum-induced interstrand cross-links [49]. In the context of bladder cancer, the de-L-lactylation of ARHGDIB K47/K50 activates the Rac1-MRN-ATM-CHK2 axis [63] and DHX15 K17 de-L-lactylation modulates RPL9 splicing, both of which contribute to cisplatin resistance [61]. XRCC1 K247 L-lactylation (Section "Genomic stability and the DNA damage response") underlies glioblastoma resistance to alkylating agents [82], and PARP1 K654 L-lactylation (Section "Genomic stability and the DNA damage response") underlies resistance in esophageal squamous cell carcinoma [69, 156]. NBS1 K388 L-lactylation (Section "Genomic stability and the DNA damage response"), together with L-lactylation of multiple Ku70/Ku80 sites, supports radiotherapy resistance in lung adenocarcinoma [17, 52], while RPA1 K88/K163/K167/K267 L-lactylation (Section "Genomic stability and the DNA damage response") further contributes to radiotherapy resistance [48]. Furthermore, eEF1A2 K408 (Section "Oncogenic signaling and phenotypic plasticity") and BLM K24 (Section "Genomic stability and the DNA damage response") L-lactylation jointly promote resistance to anthracycline and topoisomerase inhibitors [18, 85]. However, there have been reports of PPP1R11 L-lactylation stabilizing a protein and facilitating the ubiquitin-mediated degradation of MDR2, thereby reducing drug efflux and sensitizing tumor cells to chemotherapy [192]. Collectively, these modifications establish a mechanistic link between metabolic stress, lactylation, and adaptive genotoxic resistance across multiple tumor contexts.

### Resistance to targeted and endocrine therapies

Resistance to targeted therapies generally arises from bypass activation or the stabilization of downstream effectors [193–195]. In EGFR-mutated lung adenocarcinoma cells, treatment-induced metabolic stress triggers ENO1 L-lactylation at K89, which sustains high glycolytic flux and promotes intrinsic resistance to EGFR-TKIs [19]. Pharmacologically blocking lactate production or neutralizing ENO1 effectively restored drug sensitivity [19]. L-lactylation of the mitochondrial Mic60 at K282 preserves cristae structure and OXPHOS efficiency, contributing to resistance to EGFR-TKIs and metastasis [113]. In the context of melanoma, LSD1 K503 L-lactylation blocks its TRIM21-mediated degradation and suppresses TFRC transcription, inhibiting ferroptosis and ensuring resistance to BRAF/MEKis [88]. Multikinase inhibitor resistance in the context of hepatocellular carcinoma is mediated by ABCF1 K430 L-lactylation, which increases HIF-1α expression, glycolysis, and angiogenesis [36]. Furthermore, AARS1-catalyzed AKR1B10 K173 L-lactylation and IGF2BP3 K76 L-lactylation stabilize glycolysis and antioxidant pathways [53, 196]. In colorectal cancer, ZNF207-driven PRDX1 L-lactylation activates NRF2, promoting regorafenib resistance [159].

Endocrine therapy resistance emerges via the induction of stemness and alternative splicing. ZMIZ1 L-lactylation increases Nanog expression and cholesterol uptake by ER⁺ breast cancer cells, resulting in resistance to tamoxifen [128]. Furthermore, KDM5B-mediated lactate accumulation drives hnRNPA1 K179la and SNRPA L-lactylation in prostate cancer cells, stabilizing splicing regulators to generate AR-V7 variants and thereby conferring resistance to enzalutamide and inducing androgen deprivation [39, 197].

Collectively, these mechanisms establish L-lactylation as a multitiered adaptive switch that integrates metabolic reprogramming, ferroptosis evasion, and epigenetic plasticity to drive pan-cancer therapy resistance.

### Resistance to immunotherapy

L-Lactate critically blunts the efficacy of immune checkpoint blockade (ICB) and adoptive cell therapies by inducing profound immunosuppressive modifications within the tumor microenvironment [22, 86, 87, 93, 115, 198]. Targeted evasion of proteasomal degradation via L-lactylation has emerged as a central paradigm of ICB resistance. Most notably, PD-L1 K271/K280 L-lactylation (Section "Regulation of the tumor microenvironment") drives ICB failure in gastric cancer and other malignancies by sustaining checkpoint-ligand surface expression under therapeutic pressure [87, 177, 178]. Beyond immune checkpoints, L-lactylation reprograms specific immune populations. L-lactylation of MOESIN at K72 activates TGF-β signaling in regulatory T cells (Tregs), increasing their immunosuppressive activity [179], whereas L-lactylation of APOC2 at K70 stimulates the release of free fatty acids to recruit Tregs and induce PD-1 inhibitor resistance in the context of lung cancer [182]. METTL3 K281/K345 L-lactylation (Section "Regulation of the tumor microenvironment") reinforces the same STAT3–IL-6/IL-10/iNOS myeloid program under immunotherapy, contributing to ICB resistance [41]. During adoptive cell therapy, CD44 K158 L-lactylation on CD8⁺ T cells in KRAS-mutated colorectal cancer models disrupts hyaluronic acid binding and blocks downstream AKT signaling, decreasing CAR-T-cell therapeutic efficacy [86]. L-lactylation of ENSA at K63 in pancreatic cancer cells polarizes TAMs via SRC/STAT3/CCL2 signaling [22, 198]. Finally, L-lactylation impairs innate immune sensing: STAT1 K193 L-lactylation (Section "Regulation of the tumor microenvironment") similarly blunts interferon-driven chemokine (CXCL9/10/11) output, limiting the T-cell influx required for an ICB response [93, 115].

## Clinical prospects and future directions

Non-histone lysine L-lactylation constitutes a spatially compartmentalized, highly dynamic regulatory layer that convergently mobilizes under therapeutic stress. Pharmacologically inhibiting the L-lactylation cascade presents a profound opportunity to resensitize tumors to multiple treatment modalities.

### Hierarchical biomarkers: From global to site-specific L-lactylomes

L-lactylation-based signatures offer a promising translational avenue for predicting tumor adaptation beyond static genomic or transcriptomic readouts [199–202]. While total L-lactylation levels correlate with gross lactate accumulation and advanced stage in patients with colorectal cancer [203], NSCLC [204], hepatocellular carcinoma [4], and gastric cancer [1, 21], they ultimately lack functional specificity [205, 206]. In contrast, site-specific L-lactylation affords critical mechanistic resolution. For instance, mass spectrometry has mapped > 9,000 modification sites in HCC cells [117], including MOESIN L-lactylation at K72 which is linked to Treg-mediated immunosuppression [179]. Under therapeutic pressure, these precise site-specific alterations have been applied to accurately predict adaptive behaviors, such as cetuximab resistance in individuals with colorectal cancer [207, 208] and hypoxia-induced resistance in individuals with HCC [36, 209, 210]. Currently, clinical translation is constrained by low-throughput mass spectrometry approaches and the scarcity of site-specific, stereochemically validated antibodies. Nevertheless, integrating L-lactylation-associated transcriptomic signatures has allowed the successful stratification of patients with endometrial cancer [98], cervical cancer [147], and lung adenocarcinoma [11], establishing a hierarchical biomarker framework for scalable clinical application.

### Pharmacological intervention

**Fig. 8:**
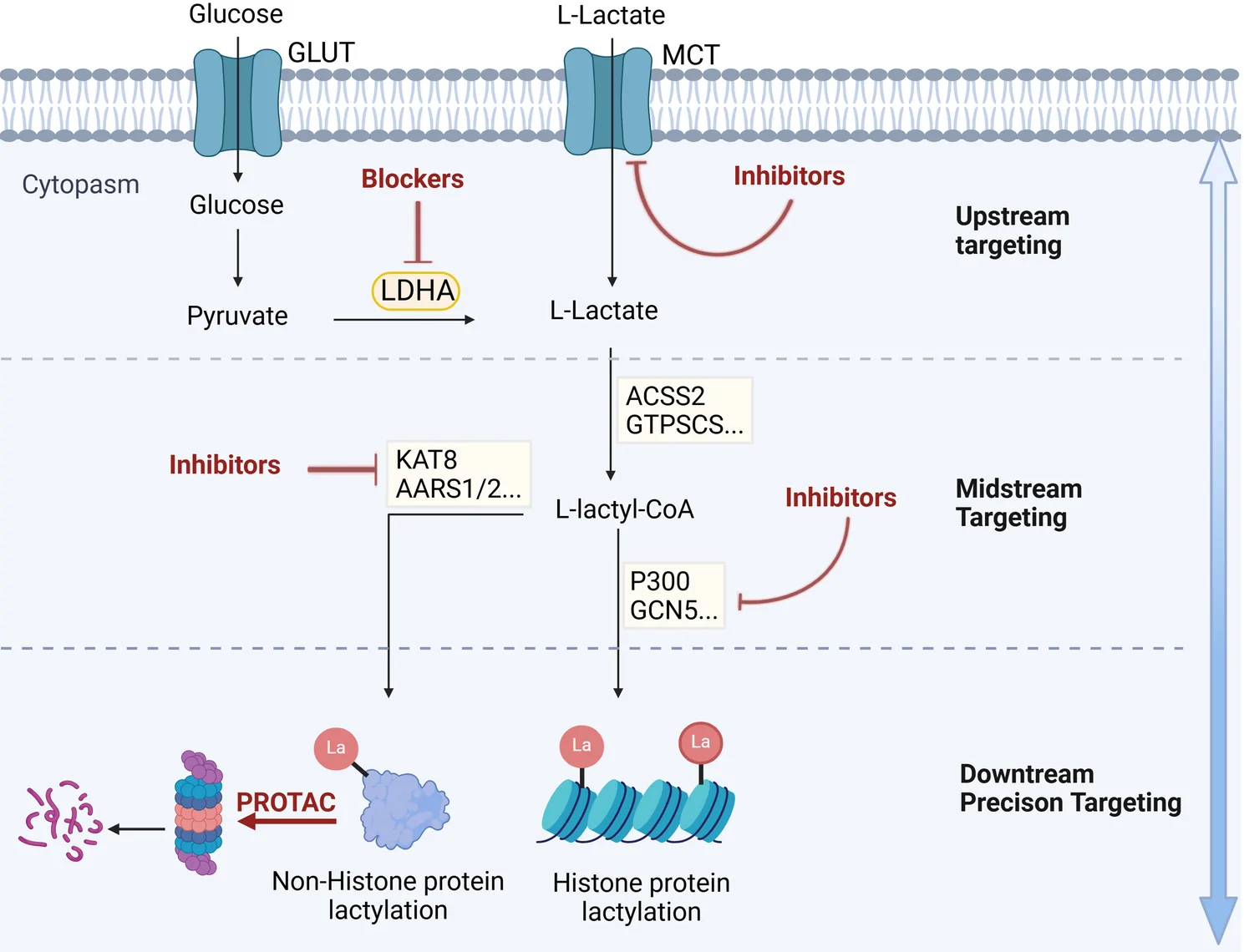
Hierarchical therapeutic strategies targeting the L-lactylation cascade. Upstream interventions block lactate generation (LDHA inhibitors) or transmembrane transport (MCT inhibitors) to deplete the precursor pool. Midstream strategies target the catalytic machinery, including lactyl-CoA synthetases (ACSS2, GTPSCS) and lactyltransferases (EP300, GCN5, AARS1/2), to prevent modification deposition. Downstream precision targeting interferes with specific pathogenic L-lactylation events, utilizing proteolysis-targeting chimeras (PROTACs) or interface mimetics. Importantly, integrating these targeted pharmacological approaches with dietary restriction and stereoisomer-resolved chemobiological probes offers a comprehensive strategy to overcome adaptive resistance and maximize immunotherapy efficacy

Therapeutic strategies can be conceptualized along the lactate–writer/eraser–functional target axis (Table S3) (Fig. 8). Upstream targeting of lactate generation (e.g., LDHA inhibitors) or transport (MCT1/4 blockers) diminishes tumor lactate pools and suppresses growth or angiogenesis [211–217]; however, broad systemic toxicity and metabolic compensation remain significant clinical barriers [218–223]. Midstream strategies, including EP300/CBP inhibitors, target catalytic enzymes to suppress substrate L-lactylation and downstream transcription [19, 38, 224] or conceptually target the AARS1/2 sensor [53]. A critical limitation of this strategy is pleiotropy, as EP300/CBP and sirtuin/HDAC erasers also regulate acetylation and other acyl modifications, leading to severe off-target epigenetic toxicity [225–229]. Downstream approaches offer greater precision by aiming to selectively inhibit specific functional L-lactylation events, such as by blocking modified protein‒protein interfaces or inducing targeted degradation. Emerging technologies, including computationally designed interface mimetics [217, 230] and PROTACs [231, 232], provide conceptual frameworks for modification-specific targeting, although designing high-affinity ligands capable of exclusively recognizing L-lactylated moieties remains a formidable challenge in structural biology.

### Rational combination therapies

In the near term, combinatorial strategies targeting distinct nodes of the L-lactylation cascade offer the most pragmatic approach to overcoming adaptive therapeutic resistance. Upstream interventions include combining ENO1-neutralizing antibodies or oxamate with osimertinib to restore sensitivity in patients with EGFR-mutant lung adenocarcinoma [19]. Similarly, pairing cisplatin with glycolysis inhibitors (e.g., 2-deoxy-D-glucose) significantly reduces the level of intracellular lactate, attenuates HK2 K873 L-lactylation-driven glycolysis, and resensitizes non-small cell lung cancer cells to chemotherapy [102]. Crucially, metabolic reprogramming via dietary interventions, such as specific amino acid restrictions, fundamentally alters the L-lactylation status of immune checkpoints, offering a potent, nonpharmacological strategy that strongly synergizes with anti-PD-1 immunotherapy [179]. Midstream combinations include pairing EP300 inhibition (e.g., A-485) with enzalutamide to block KDM5B-driven hnRNPA1 K179 L-lactylation and AR-V7 splicing in the context of prostate cancer [39]. Additionally, HDAC inhibition (e.g., chidamide) plus cisplatin disrupts HDAC2-mediated METTL3 de-L-lactylation, strongly increasing its cytotoxicity in the context of triple-negative breast cancer [42]. Downstream interventions, such as the use of saikosaponin D to increase MRE11 K673 de-L-lactylation or AARS1 inhibition to prevent PKM2 K206 L-lactylation-mediated vasculogenic mimicry, could significantly improve the therapeutic response [43, 141].

### Challenges with clinical translation and future directions

A key prerequisite for future clinical translation lies in the precise stereochemical resolution of enzymatically driven L-lactylation from nonenzymatic D-lactylation artifacts [24, 27]. Currently, the field is hindered by limited in vivo validation methodologies [233, 234], profound spatiotemporal tumor heterogeneity [235], and a lack of single-cell resolution technologies capable of mapping PTMs [188]. Critical priorities must include the discovery of L-lactylation-specific reader proteins, the development of dynamic detection probes, and rigorous in vivo causal validation of high-confidence targets (e.g., MRE11 K673, NBS1 K388, and PD-L1 modifications) [188, 236]. Ultimately, while the concept of a definitive “lactylation code” remains premature, non-histone L-lactylation is increasingly recognized as a metabolically responsive regulatory layer. Future translational success will depend on advancing stereoisomer-resolved chemobiological tools, shifting the paradigm from blunt metabolic inhibition toward precise, modification state-specific therapeutic interventions.

## Supplementary Information

Supplementary Material 1.

Supplementary Material 2.

Supplementary Material 3.

## Data Availability

No datasets were generated or analysed during the current study.

## Abbreviations

EP300: E1A binding protein EP300
CBP: CREB-binding protein
AARS1: Alanyl-tRNA synthetase 1
AARS2: Alanyl-tRNA synthetase 2
DNA: Deoxyribonucleic acid
Kla: Lysine lactylation
HCC: Hepatocellular carcinoma
PPIs: Protein–protein interactions
RNA: Ribonucleic acid
DDR: DNA damage repair
KL-la: Lysine L-lactylation
KD-la: Lysine D-lactylation
Kce: N-ε-(carboxyethyl)-lysine
MS/MS: Tandem mass spectrometry
K→R: Lysine-to-arginine
K→Q: Lysine-to-glutamine
CoA: Coenzyme A
AMP: Adenosine monophosphate
HAT: Histone acetyltransferase
Kac: Lysine acetylation
KAT3: Lysine acetyltransferase 3
ABCF1: ATP binding cassette subfamily F member 1
NUSAP1: Nucleolar and spindle associated protein 1
HNRNPA1: Heterogeneous nuclear ribonucleoprotein A1
NCL: Nucleolin
METTL3: Methyltransferase 3
MRE11: Meiotic recombination 11
YTHDC1: YTH domain-containing protein 1
NMNAT1: Nicotinamide nucleotide adenylyltransferase 1
GNAT: GCN5-related N-acetyltransferase
HAT1: Histone acetyltransferase 1
RPA1: Replication protein A1
GCN5: General control non-derepressible 5
XLF: XRCC4-like factor
KAT2A: Lysine acetyltransferase 2 A
RCC2: Regulator of chromosome condensation 2
SERBP1: SERPINE1 mRNA binding protein 1
mRNA: Messenger RNA
MYST: MOZ, Ybf2/Sas3, Sas2, Tip60
TIP60: Tat-interactive protein 60 kDa
NBS1: Nijmegen breakage syndrome 1
MRN: MRE11-RAD50-NBS1
Ku70: Ku autoantigen protein 70
Ku80: Ku autoantigen protein 80
AARS: Alanyl-tRNA synthetase
m6A: N⁶-methyladenosine
ACSS2: Acyl-CoA synthetase short-chain family member 2
GTPSCS: GTP-specific succinyl-CoA synthetase
SIRT1: Sirtuin 1
SIRT2: Sirtuin 2
SIRT3: Sirtuin 3
HDAC: Histone deacetylase
HDAC1: Histone deacetylase 1
HDAC2: Histone deacetylase 2
HDAC3: Histone deacetylase 3
NAD: Nicotinamide adenine dinucleotide
m5C: 5-methylcytosine
LDHA: Lactate dehydrogenase A
NHEJ: Non-homologous end joining
FEN1: Flap endonuclease 1
TCA: Tricarboxylic acid cycle
LLPS: Liquid–liquid phase separation
ALDOA: Aldolase A
PKM2: Pyruvate kinase M2
PFKP: Phosphofructokinase, platelet
HR: Homologous recombination
CD44: Cluster of differentiation 44
PD-L1: Programmed death-ligand 1
BLM: Bloom syndrome protein
PTBP1: Polypyrimidine tract-binding protein 1
AKR1B10: Aldo-keto reductase family 1 member B10
Axin1: Axis inhibition protein 1
FLCN: Folliculin
ENO1: Enolase 1
XRCC1: X-ray repair cross-complementing protein 1
KDM3A: Lysine demethylase 3 A
RIG-I: Retinoic acid-inducible gene I
PARP1: Poly(ADP-ribose) polymerase 1
YAP: Yes-associated protein
CRM1: Chromosome region maintenance 1
STAT1: Signal transducer and activator of transcription 1
JAK2: Janus kinase 2
RBPs: RNA-binding proteins
YBX1: Y-box binding protein 1
IDRs: Intrinsically disordered regions
HIF1A: Hypoxia-inducible factor 1 alpha
p53: Tumor protein p53
PKM1: Pyruvate kinase M1
EMT: Epithelial-mesenchymal transition
PFKM: Phosphofructokinase, muscle
SHMT2: Serine hydroxymethyltransferase 2
MTHFD1L: Methylenetetrahydrofolate dehydrogenase (NADP+ dependent) 1-like
PSMD14: Proteasome 26 S subunit, non-ATPase 14
HK2: Hexokinase 2
ALYREF: Aly/REF export factor
IGF1R: Insulin-like growth factor 1 receptor
NSUN2: NOP2/Sun RNA methyltransferase 2
TME: Tumor microenvironment
KAT8: Lysine acetyltransferase 8
MDH2: Malate dehydrogenase 2
NADH: Nicotinamide adenine dinucleotide (reduced)
ATP: Adenosine triphosphate
CS: Citrate synthase
SLC25A1: Solute carrier family 25 member 1
PPP: Pentose phosphate pathway
G6PD: Glucose-6-phosphate dehydrogenase
NADPH: Nicotinamide adenine dinucleotide phosphate (reduced)
DCBLD1: Discoidin, CUB and LCCL domain containing 1
ACAA2: Acetyl-CoA acyltransferase 2
IGF2BP3: Insulin-like growth factor 2 mRNA binding protein 3
PCK2: Phosphoenolpyruvate carboxykinase 2
Mic60: Mitochondrial inner membrane organizing system 60 kDa protein
ME2: Malic enzyme 2
FFA: Free fatty acid
SAM: S-adenosylmethionine
LC: Lung Cancer
PDAC: Pancreatic Ductal Adenocarcinoma
CRC: Colorectal Cancer
EC: Esophageal Cancer
TNBC: Triple-Negative Breast Cancer
RCC: Renal Cell Carcinoma
OC: Ovarian Cancer
CSC: Cancer stem cell
CCNE2: Cyclin E2
RHOA: Ras homolog family member A
GTP: Guanosine triphosphate
ROCK: Rho-associated coiled-coil containing protein kinase
MLC: Myosin light chain
RET: Rearranged during transfection
MAPK: Mitogen-activated protein kinase
PI3K: Phosphoinositide 3-kinase
AKT: Protein kinase B
PIK3CB: Phosphatidylinositol-4,5-bisphosphate 3-kinase catalytic subunit beta
ULK1: Unc-51 like autophagy activating kinase 1
PSMA4: Proteasome 20 S subunit alpha 4
cGAS: Cyclic GMP-AMP synthase
CEACAM6: Carcinoembryonic antigen-related cell adhesion molecule 6
TPX2: Targeting protein for Xklp2
SLC26A3: Solute carrier family 26 member 3
NME1: NME/NM23 nucleoside diphosphate kinase 1
ERK: Extracellular signal-regulated kinase
YY1: Yin and Yang 1
FBXO33: F-box protein 33
Snail1: Snail family transcriptional repressor 1
TWIST1: Twist family bHLH transcription factor 1
CENPA: Centromere protein A
ZMIZ1: Zinc finger MIZ-type containing 1
Nanog: Nanog homeobox
SP1: Specificity protein 1
CENPL: Centromere protein L
ATRX: Alpha thalassemia/mental retardation syndrome X-linked
NFIL3: Nuclear factor, interleukin 3 regulated
H3K27ac: Histone H3 lysine 27 acetylation
FTO: FTO alpha-ketoglutarate dependent dioxygenase
ALKBH5: AlkB homolog 5, RNA demethylase
EPAS1: Endothelial PAS domain protein 1
CEBPD: CCAAT enhancer binding protein delta
SOX9: SRY-box transcription factor 9
NOL6: Nucleolar protein 6
STAMBP: STAM binding protein
c-Myc: Cellular myelocytomatosis oncogene
SQLE: Squalene epoxidase
PUMA: p53 upregulated modulator of apoptosis
RBP: RNA-binding protein
RBM15: RNA binding motif protein 15
WTAP: Wilms’ tumor 1-associating protein
BCL2: B-cell lymphoma 2
E2F2: E2F transcription factor 2
MAD2L1: Mitotic arrest deficient 2 like 1
MADD: MAP kinase activating death domain
iCCA: Intrahepatic cholangiocarcinoma
G3BP2: GTPase activating protein (SH3 domain) binding protein 2
CDCP1: CUB domain containing protein 1
STC1: Stanniocalcin 1
JUND: JunD proto-oncogene
AR-V7: Androgen receptor splice variant 7
PAK6: P21 (RAC1) activated kinase 6
PKM: Pyruvate kinase M
ESRP1: Epithelial splicing regulatory protein 1
circPHGDH: Circular RNA phosphoglycerate dehydrogenase
miR-149: MicroRNA 149
RAP1B: RAP1B, member of RAS oncogene family
YTHDF3: YTH N6-methyladenosine RNA binding protein 3
KDM6B: Lysine demethylase 6B
DHX15: DEAH-box helicase 15
eEF1A2: Eukaryotic translation elongation factor 1 alpha 2
eEF2: Eukaryotic translation elongation factor 2
Oct4: Octamer-binding transcription factor 4
Sox2: SRY-box transcription factor 2
FOSL1: FOS like 1
SNAI2: Snail family transcriptional repressor 2
MYC: MYC proto-oncogene
MAZ: MYC associated zinc finger protein
ANTXR1: ANTXR cell adhesion molecule 1
RhoC: Ras homolog family member C
ROCK1: Rho associated coiled-coil containing protein kinase 1
SMAD5: SMAD family member 5
PFKFB4: 6-phosphofructo-2-kinase/fructose-2,6-biphosphatase 4
UPF1: UPF1 RNA helicase and ATPase
SPTAN1: spectrin alpha, non-erythrocytic 1
NOTCH1: Notch receptor 1
HES1: Hes family bHLH transcription factor 1
HuR: ELAV like RNA binding protein 1
CUGBP1: CUG triplet repeat, RNA binding protein 1
CD133: Prominin 1
E-cadherin: Cadherin 1
CTCF: CCCTC-binding factor
TERF2: Telomeric repeat binding factor 2
POLR2A: RNA polymerase II subunit A
Wnt: Wingless-type MMTV integration site family
CFL1: Cofilin 1
PRMT1: Protein arginine methyltransferase 1
PPP1R14B: Protein phosphatase 1 regulatory inhibitor subunit 14B
BER: Base excision repair
SSBR: Single-strand break repair
ssDNA: Single-stranded DNA
DNA-PKcs: DNA-dependent protein kinase catalytic subunit
LIG4: DNA ligase 4
ARHGDIB: Rho GDP-dissociation inhibitor 2
Rac1: Ras-related C3 botulinum toxin substrate 1
ATM: Ataxia telangiectasia mutated
CHK2: Checkpoint kinase 2
RAD51: DNA repair protein RAD51 homolog 1
GBM: Glioblastoma
LUAD: Lung adenocarcinoma
GC: Gastric cancer
BC: Bladder cancer
OEA: Ovarian endometrioid adenocarcinoma
GPX4: Glutathione peroxidase 4
GCLM: Glutamate-cysteine ligase modifier subunit
GSH: Glutathione
PCBP2: Poly(rC) binding protein 2
PRDX1: Peroxiredoxin 1
NRF2: Nuclear factor erythroid 2-related factor 2
FSP1: Ferroptosis suppressor protein 1
SPRING: SREBP1-induced growth protein
LSD1: Lysine-specific demethylase 1 A
TRIM21: Tripartite motif-containing protein 21
TFRC: Transferrin receptor
SUMO2: Small ubiquitin-like modifier 2
ACSL4: Acyl-CoA synthetase long-chain family member 4
GCLC: Glutamate-cysteine ligase catalytic subunit
LDHB: Lactate dehydrogenase B
SLC7A11: Solute carrier family 7 member 11
PRMT5: Protein arginine methyltransferase 5
NF-κB: Nuclear factor kappa B
NLRP1: NLR family pyrin domain containing 1
GSDMD: Gasdermin D
IL-1β: Interleukin-1 beta
CNPY3: Canopy FGF signaling regulator 3
NUDT21: Nudix hydrolase 21
FDX1: Ferredoxin 1
CFIm: Cleavage factor Im
3′UTR: 3′ untranslated region
METTL16: Methyltransferase-like protein 16
DLAT: Dihydrolipoamide S-acetyltransferase
mTORC1: Mechanistic target of rapamycin complex 1
TFEB: Transcription factor EB
Vps34: Vacuolar protein sorting 34
ATG14L: Autophagy-related 14-like protein
UVRAG: UV radiation resistance-associated gene protein
mTOR: Mechanistic target of rapamycin
FOXO3: Forkhead box O3
CASP3: Caspase 3
ACF1: ATP-utilizing chromatin assembly and remodeling factor 1
H3K4me3: Histone H3 lysine 4 trimethylation
ABHD6: Abhydrolase domain containing 6
FIS1: Fission, mitochondrial 1
DRP1: Dynamin-related protein 1
ROS: Reactive oxygen species
CA3: Carbonic anhydrase 3
DUOX2: Dual oxidase 2
CD8: Cluster of differentiation 8
FERM: Band 4.1, ezrin, radixin, moesin homology domain
TGF-β: Transforming growth factor beta
SMAD3: SMAD family member 3
Treg: Regulatory T cell
PGE2: Prostaglandin E2
Rac2: Ras-related C3 botulinum toxin substrate 2
MDSC: Myeloid-derived suppressor cell
APOC2: Apolipoprotein C2
ENSA: Endosulfine alpha
SRC: SRC proto-oncogene, non-receptor tyrosine kinase
STAT3: Signal transducer and activator of transcription 3
CCL2: C-C motif chemokine ligand 2
TAM: Tumor-associated macrophage
EGR1: Early growth response protein 1
IKZF1: IKAROS family zinc finger 1
STING: Stimulator of interferon genes
CXCL9: C-X-C motif chemokine ligand 9
CXCL10: C-X-C motif chemokine ligand 10
CXCL11: C-X-C motif chemokine ligand 11
IL-6: Interleukin 6
IL-10: Interleukin 10
iNOS: Inducible nitric oxide synthase
CD276: Cluster of differentiation 276
NECTIN2: Nectin cell adhesion molecule 2
VIRMA: Vir-like m6A methyltransferase associated
COX2: Cyclooxygenase 2
VEGFA: Vascular endothelial growth factor A
ASH2L: ASH2 like histone lysine methyltransferase complex subunit
MLL: Mixed lineage leukemia
H3K4: Histone H3 lysine 4
KIAA1199: KIAA1199 protein (cell migration inducing hyaluronidase 1)
VE-cadherin: Vascular endothelial cadherin
EphA2: EPH receptor A2
sema3A: Semaphorin 3 A
VEGFR2: Vascular endothelial growth factor receptor 2
Rab7A: Ras-related protein Rab-7a
RPL9: Ribosomal protein L9
PPP1R11: Protein phosphatase 1 regulatory subunit 11
MDR2: Multidrug resistance protein 2
EGFR: Epidermal growth factor receptor
EGFR-TKIs: Epidermal growth factor receptor tyrosine kinase inhibitors
OXPHOS: Oxidative phosphorylation
BRAF: B-Raf proto-oncogene, serine/threonine kinase
MEKi: Mitogen-activated protein kinase kinase inhibitor
ZNF207: Zinc finger protein 207
ER: Estrogen receptor
SNRPA: Small nuclear ribonucleoprotein polypeptide A
ICB: Immune checkpoint blockade
KRAS: Kirsten rat sarcoma viral oncogene homolog
CAR-T: Chimeric antigen receptor T-cell
NSCLC: Non-small cell lung carcinoma
MCT1: Monocarboxylate transporter 1
MCT4: Monocarboxylate transporter 4
PROTAC: Proteolysis-targeting chimera
MCT: Monocarboxylate transporter
PTM: Post-translational modification
