# Supplementary material for: Non-histone protein L-lactylation in cancer: a metabolic switch for immune evasion and therapeutic resistance
Source: Mol Cancer. 2026 Aug 1;25:259. doi: 10.1186/s12943-026-02759-7 (PMC13721481; doi:10.1186/s12943-026-02759-7)
Supplement: Supplementary file 1 [file 12943_2026_2759_MOESM1_ESM.docx]

**Table 1** **Summary of Reported Non-Histone Lactylation Modification Events in Cancer**

| **Substrate Protein** | **Modification Site** | **Writer** | **Eraser** | **Regulatory Mode** | **Functional Direction** | **Downstream Effect Level** | **Cancer Type** | **Drug Resistance Relevance** | **Validation Method** | **Refere-nce** |
| --- | --- | --- | --- | --- | --- | --- | --- | --- | --- | --- |
| ABCF1  (Q8NE71) | K430 | P300 | HDAC1, HDAC3 | Regulation of subcellular localization/Protein-Protein interactions/Modulation of nucleic acid binding affinity | Tumor promoting | Indirect transcriptional regulation | HCC | N/D | MS identification/K→  R/K→Q/in vivo model/clinical samples | Hong  et al.^36^ |
| ABHD6  (Q9BV23) | K245, K223 | N/D | N/D | Regulation of subcellular localization/Protein-Protein interactions | Tumor promoting | Direct protein regulation | HCC | Targeted | MS identification/K→ R/in vivo model/clinical samples | Sun  et al.^171^ |
| ACAA2  (P42765) | K214, K270 | N/D | N/D | Enhances catalytic activity | Tumor promoting | Direct protein regulation | TNBC | N/D | MS identification/K→ R/in vivo model/clinical samples | Cui  et al.^111^ |
| ACF1  (Q9NQ94) | N/D | NAA20 | N/D | Regulation of subcellular localization | Tumor promoting | Indirect transcriptional regulation | NB | Chemotherapy/Radiotherapy | MS identification/in vivo model/clinical samples | Han  et al.^170^ |
| AKR1B10  (O60218) | K173 | AARS1 | N/D | Regulation of protein stability/Protein-Protein interactions | Tumor promoting | Direct protein regulation, Indirect transcriptional regulation | HCC | Targeted | MS identification/K→ R/in vivo model/clinical samples | Liu  et al.^53^ |
| ALDOA  (P04075) | K147 | N/D | N/D | Modulation of enzymatic activity | Tumor promoting | Direct protein regulation | N/D | N/D | MS identification | Cheng  et al.^7^ |
| ALYREF  (Q86V81) | K171 | N/D | N/D | Regulation of protein stability | Tumor promoting | Direct protein regulation | LSCC | N/D | MS identification/K→ R/in vivo model/clinical samples | Bian  et al.^101^ |
| ANTXR1  (Q9H6X2) | K453 | KAT8 | N/D | Regulation of protein stability/Protein-Protein interactions | Tumor promoting | Direct protein regulation | CRC | Chemotherapy | MS identification/K→ R/in vivo model/clinical samples | He  et al.^140^ |
| APOC2  (P02655) | K70 | P300 | HDAC3 | Regulation of protein stability/Protein-Protein interactions | Tumor promoting | Direct protein regulation | NSCLC | Immunotherapy | MS identification/K→ R/in vivo model/clinical samples | Chen  et al.^182^ |
| ASH2L  (Q9UBL3) | K312 | AARS1 | HDAC1 | Modulation of nucleic acid binding affinity | Tumor promoting | Indirect transcriptional regulation | HCC | N/D | MS identification/K→ R/in vivo model/clinical samples | Han  et al.^58^ |
| Axin1  (O15169) | K147 | N/D | N/D | Regulation of protein stability | Tumor promoting | Direct protein regulation | ESCA | N/D | K→R/in vivo model/IP/WB | Li  et al.^89^ |
| BLM  (P54132) | K24 | AARS1 | N/D | Regulation of protein stability/Protein-Protein interactions | Tumor promoting | Direct protein regulation | BLCA/BC/HCC | Chemotherapy | MS identification/K→ R/in vivo model/clinical samples | Li  et al.^18^ |
| CASP3  (P42574) | K14 | N/D | N/D | Modulation of enzymatic activity | Tumor promoting | Direct protein regulation | ALL | Chemotherapy | MS identification/K→ R/in vivo model/clinical samples | Lin  et al.^169^ |
| CCNE2  (O96020) | K347 | N/D | SIRT3 | N/D | Tumor promoting | Direct protein regulation | HCC | N/D | MS identification/K→ R/K→Q/in vivo model/clinical samples | Jin  et al.^117^ |
| CD44  (P16070) | K158 | AARS1 | N/D | Protein-Protein interactions | Tumor promoting | Direct protein regulation | CRC | immunotherapy | MS identification/K→ R/in vivo model/clinical samples | Yang  et al.^86^ |
| CENPA  (P49450) | K124 | ND | ND | Regulation of subcellular localization / Protein-Protein interactions /Modulation of nucleic acid binding affinity | Tumor promoting | Indirect transcriptional regulation | HCC | N/D | K→R/in vivo model/clinical samples | Liao  et al.^126^ |
| cGAS  (Q8N884) | K21 | N/D | N/D | Regulation of protein stability/Regulation of subcellular localization/Protein-Protein interactions | Tumor promoting | Direct protein regulation | LUAD | N/D | MS identification/K→ R/in vivo model/clinical samples | Rao  et al.^115^ |
| c-Myc  (P01106) | N/D | N/D | N/D | Regulation of protein stability | Tumor promoting | Direct protein regulation | HCC | N/D | qPCR/WB/Co-IP | Yao  et al.^142^ |
| DCBLD1  (Q8N8Z6) | K172, K497, K486, K167 | N/D | N/D | Regulation of protein stability | Tumor promoting | Direct protein regulation | CECA | N/D | MS identification/K→ A/in vivo model/clinical samples | Meng  et al.^110^ |
| eEF1A2  (Q05639) | K408 | KAT8 | N/D | Modulation of enzymatic activity | Tumor promoting | Direct protein regulation | CRC | N/D | MS identification/K→ R/in vivo model/clinical samples | Xie  et al.^85^ |
| eEF2  (P13639) | K439, K594 | N/D | N/D | Modulation of enzymatic activity | Tumor promoting | Direct protein regulation | CRC | N/D | MS identification/K→R | Rana  et al.^139^ |
| EGR1  (Q29W20) | K422 | N/D | N/D | Regulation of subcellular localization | Tumor promoting | Indirect transcriptional regulation | ICC | Immunotherapy | K→R/in vivo model/clinical samples | Liang  et al.^183^ |
| ENO1  (P00924) | K64, K89, K262 | EP300 | N/D | Regulation of protein stability/Modulation of enzymatic activity | Tumor promoting | Direct protein regulation | TNBC | N/D | MS identification/K→  R/in vivo model/clinical samples | Cheng  et al.^90^ |
| ENO1  (P00924) | K89 | P300, CBP | N/D | Modulation of enzymatic activity/Protein-Protein interactions | Tumor promoting | Direct protein regulation | LUAD | Targeted | MS identification/in vivo model/clinical samples | Gan  et al.^19^ |
| ENSA  (O43768) | K63 | EP300 | N/D | Protein-Protein interactions/Modulation of enzymatic activity | Tumor promoting | Indirect transcriptional regulation | PDAC | Immunotherapy | MS identification/K→  R/in vivo model/clinical samples | Sun  et al.^198^ |
| ENSA  (O43768) | K63 | EP300 | N/D | Protein-Protein interactions/Modulation of enzymatic activity | Tumor promoting | Indirect transcriptional regulation | PDAC | Immunotherapy | MS identification/K→  R/in vivo model/clinical samples | Chen  et al.^22^ |
| ERK1  (P27361) | K248 | GCN5 | N/D | Modulation of enzymatic activity/Protein-Protein interactions | Tumor promoting | Direct protein regulation/Indirect transcriptional regulation | NSCLC/CRC/GBM/TNBC | Chemotherapy | MS identification/K→  R/in vivo model/clinical samples | Huang et al.^123^ |
| ERK2  (P28482) | K231 | GCN5 | N/D | Modulation of enzymatic activity/Protein-Protein interactions | Tumor promoting | Direct protein regulation/Indirect transcriptional regulation | NSCLC/CRC/GBM/TNBC | Chemotherapy | MS identification/K→  R/in vivo model/clinical samples | Huang et al.^123^ |
| ESRP1  (Q6NXG1) | K43 | N/D | N/D | Regulation of protein stability | Tumor promoting | Direct protein regulation | PCA | N/D | K→R/IP/WB | Wang  et al.^137^ |
| FEN1  (P39748) | K345 | N/D | N/D | Protein-Protein interactions | Tumor promoting | Indirect transcriptional regulation | LUAD | Chemotherapy | K→R/IP/WB | Li  et al.^52^ |
| FLCN  (Q8NFG4) | K523 | AARS1 | Sirt1 | Protein-Protein interactions/Regulation of subcellular localization | Tumor promoting | Direct protein regulation/Indirect transcriptional regulation | CRC | N/D | MS identification/K→  R/in vivo model/clinical samples | Zhu  et al.^55^ |
| FOXO3  (O43524) | K259, K262, K269, K270 | N/D | N/D | N/D | Tumor promoting | Indirect transcriptional regulation | PDAC | N/D | MS identification/Co-IP/in vivo model/clinical samples | Gong  et al.^168^ |
| G6PD  (P11413) | K45 | N/D | N/D | Modulation of enzymatic activity | Tumor promoting | Direct protein regulation | CECA/LC | N/D | MS identification/K→  R/K→Q/in vivo model | Meng  et al.^109^ |
| G6PD  (P11413) | K46, K47 | N/D | N/D | Modulation of enzymatic activity | Tumor promoting | Direct protein regulation | LC | N/D | K→R/in vivo model | Zhang  et al.^107^ |
| GCLM  (P48507) | K34 | ACAT2 | N/D | Modulation of enzymatic activity/Protein-Protein interactions | Tumor promoting | Direct protein regulation | PC/CRC | N/D | MS identification/K→  R/in vivo model/clinical samples | Chen  et al.^160^ |
| GPX4  (P36969) | N/D | N/D | N/D | Regulation of protein stability | Tumor promoting | Direct protein regulation | GC | N/D | Co-IP | Hu  et al.^157^ |
| HAT1  (O14929) | K15 | HAT1 | N/D | Modulation of enzymatic activity | Tumor promoting | Direct protein regulation | GBM/LUAD | Radiotherapy | MS identification/K→R | Zhao  et al.^47^ |
| HAT1  (O14929) | K15 | HAT1 | N/D | Modulation of enzymatic activity | Tumor promoting | Direct protein regulation | GBM/LUAD | Radiotherapy | MS identification/K→R | He  et al.^48^ |
| HDAC1  (Q13547) | K412 | N/D | N/D | Regulation of protein stability/Modulation of enzymatic activity | Tumor promoting | Indirect transcriptional regulation | CRC | N/D | MS identification/K→  R/in vivo model/clinical samples | Yang  et al.^65^ |
| HDAC1  (Q13547) | K200 | N/D | N/D | Protein-Protein interactions | Tumor promoting | Indirect transcriptional regulation | GBM | Chemotherapy | MS identification/K→  R/in vivo model/clinical samples | Gao  et al.^66^ |
| HIF1α  (Q16665) | N/D | N/D | N/D | Regulation of protein stability | Tumor promoting | Indirect transcriptional regulation | PCA | N/D | WB/ | Luo  et al.^188^ |
| HK2  (P52789) | K873 | N/D | N/D | Regulation of protein stability/Modulation of enzymatic activity | Tumor promoting | Direct protein regulation | NSCLC | Chemotherapy | MS identification/K→  R/in vivo model/clinical samples | Zeng  et al.^102^ |
| HNRNPA1  (P09651) | K350 | P300 | N/D | Protein-Protein interactions/Regulation of protein stability/Modulation of nucleic acid binding affinity | Tumor promoting | Indirect transcriptional regulation | BLCA | N/D | MS identification/K→  R/in vivo model/clinical samples | Wang  et al.^38^ |
| hnRNPA1  (P09651) | K179 | P300 | HDAC1,HDAC2 | Regulation of protein stability/Protein-Protein interactions | Tumor promoting | Indirect transcriptional regulation | PCA | Targeted | MS identification/K→  R/in vivo model/clinical samples | Sun  et al.^39^ |
| HNRNPC  (P07910) | K176 | N/D | N/D | Regulation of protein stability//Modulation of nucleic acid binding affinity | Tumor promoting | Indirect transcriptional regulation | PC | N/D | MS identification/K→  R/in vivo model/clinical samples | Li  et al.^136^ |
| IGF1R  (P08069) | N/D | N/D | N/D | Regulation of protein stability/Protein-Protein interactions | Tumor promoting | Direct protein regulation | LC | N/D | IP/Co‑IP/CHX | Zhang  et al.^103^ |
| IGF2BP3  (O00425) | K76 | PARK7 | N/D | Protein-Protein interactions/Regulation of protein stability | Tumor promoting | Indirect transcriptional regulation | HCC | Targeted/Chemotherapy | MS identification/K→  R/in vivo model/clinical samples | Lu  et al.^112^ |
| IGF2BP3  (O00425) | K76 | PARK7 | N/D | Protein-Protein interactions/Regulation of protein stability | Tumor promoting | Indirect transcriptional regulation | HCC | Targeted/Chemotherapy | MS identification/K→  R/in vivo model/clinical samples | Zhu  et al.^196^ |
| IKZF1  (Q13422) | K255, K241, K429 | N/D | N/D | Regulation of subcellular localization/Protein-Protein interactions | Tumor promoting | Indirect transcriptional regulation | GBM | Chemotherapy | MS identification/K→  R/in vivo model/clinical samples | Gao  et al.^184^ |
| Ku70  (P12956) | K237, K287, K461, K539, K570, K591, K596 | N/D | N/D | Regulation of subcellular localization/Protein-Protein interactions | Tumor promoting | Direct protein regulation | LUAD | Radiotherapy | MS identification | He  et al.^11^ |
| Ku70  (P12956) | K317 | P300 | N/D | Protein-Protein interactions | Tumor promoting | Direct protein regulation | GBM | Immunotherapy | MS identification/K→  R/in vivo model/clinical samples | Yi  et al.^181^ |
| Ku70  (P12956) | N/D | N/D | N/D | Protein-Protein interactions | Tumor promoting | Direct protein regulation | LUAD | Chemotherapy | MS identification | Li  et al.^52^ |
| Ku80  (P13010) | N/D | N/D | N/D | Protein-Protein interactions | Tumor promoting | Direct protein regulation | LUAD | Chemotherapy | MS identification | Li  et al.^52^ |
| Ku80  (P13010) | K543 | N/D | N/D | Protein-Protein interactions/Regulation of subcellular localization | Tumor promoting | Direct protein regulation | LUAD | Radiotherapy | MS identification | He  et al.^11^ |
| LDHA  (P00338) | K81, K318 | AARS1 | SIRT2 | Modulation of enzymatic activity | Tumor promoting | Direct protein regulation | LUAD | Chemotherapy | MS identification/K→R | Li  et al.^52^ |
| LDHB  (P07195) | K58, K156 | P300 | HDACs | Modulation of enzymatic activity/Protein-Protein interactions | Tumor promoting | Direct protein regulation | GC | N/D | MS identification/K→  R/in vivo model/clinical samples | Xiao  et al.^114^ |
| LSD1  (O60341) | K503 | N/D | N/D | Protein-Protein interactions/Regulation of protein stability/Regulation of subcellular localization | Tumor promoting | Indirect transcriptional regulation | MEL | Targeted | MS identification/K→  R/in vivo model/clinical samples | Li  et al.^88^ |
| MDH2  (P40926) | K239 | KAT8 | SIRT3 | Modulation of enzymatic activity/Protein-Protein interactions | Tumor promoting | Direct protein regulation | RCC | N/D | MS identification/K→  R/in vivo model/clinical samples | Tang  et al.^71^ |
| ME2  (P23368) | K352 | N/D | SIRT3 | Modulation of enzymatic activity | Tumor promoting | Direct protein regulation | CRC | N/D | MS identification/K→  R/in vivo model/clinical samples | Li  et al.^72^ |
| METTL16  (Q86W50) | K269 | N/D | N/D | Modulation of enzymatic activity/Regulation of protein stability | Tumor promoting | Indirect transcriptional regulation | PDAC | Immunotherapy | MS identification/K→  R/in vivo model/clinical samples | Liu  et al.^20^ |
| METTL3  (Q86U44) | K27 | P300 | HDAC2 | Protein-Protein interactions | Tumor promoting | Indirect transcriptional regulation | TNBC | Chemotherapy | MS identification/K→  R/in vivo model/clinical samples | He  et al.^42^ |
| METTL3  (Q86U44) | K281, K345 | P300 | N/D | Protein-Protein interactions | Tumor promoting | Indirect transcriptional regulation | CRC/MEL/LC | N/D | MS identification/K→R | Xiong  et al.^41^ |
| Mic60  (Q16891) | K282 | N/D | Dapk2 | Regulation of subcellular localization/Regulation of protein stability | Tumor promoting | Direct protein regulation | NSCLC | Targeted | MS identification/K→  R/K→Q/in vivo model/clinical samples | Zheng  et al.^113^ |
| MOESIN  (P26038) | K72 | N/D | N/D | Protein-Protein interactions | Tumor promoting | Indirect transcriptional regulation | HCC | Immunotherapy | MS identification/K→  R/in vivo model/clinical samples | Gu  et al.^179^ |
| MRE11  (P49959) | K673 | CBP | SIRT1, SIRT2 | Modulation of enzymatic activity/Protein-Protein interactions/Modulation of nucleic acid binding affinity | Tumor promoting | Direct protein regulation | CC/BC/TNBC | Chemotherapy/Radiotherapy | MS identification/K→  R/in vivo model/clinical samples | Chen  et al.^44^ |
| MRE11  (P49959) | K673 | CBP | HDAC5 | Modulation of enzymatic activity/Protein-Protein interactions/Modulation of nucleic acid binding affinity | Tumor promoting | Direct protein regulation | CC/BC/TNBC | Chemotherapy/Radiotherapy | MS identification/K→  R/in vivo model/clinical samples | Li  et al.^43^ |
| NBS1  (O60934) | K388 | TIP60 | HDAC3 | Protein-Protein interactions | Tumor promoting | Direct protein regulation | GC | Chemotherapy | MS identification/K→  R/in vivo model/clinical samples | Chen  et al.^17^ |
| NCL  (P19338) | K477 | P300 | N/D | Regulation of subcellular localization/Modulation of enzymatic activity | Tumor promoting | Indirect transcriptional regulation | ICC | N/D | MS identification/K→  R/in vivo model/clinical samples | Yang  et al.^40^ |
| NME1  (P15531) | K49 | N/D | N/D | Modulation of enzymatic activity | Tumor promoting | Direct protein regulation | Glioma | N/D | MS identification/K→R | Batsios et al.^116^ |
| NMNAT1  (Q9HAN9) | K128 | EP300 | HDAC1 | Regulation of subcellular localization/Modulation of enzymatic activity | Tumor promoting | Direct protein regulation | PDAC | N/D | MS identification/K→  R/in vivo model/clinical samples | Huang et al.^46^ |
| NOL6  (Q9H6R4) | K54 | N/D | N/D | Regulation of protein stability | Tumor promoting | Direct protein regulation | CRC | N/D | MS identification/K→R | Zhang  et al.^133^ |
| NSUN2  (Q08J23) | K356 | N/D | N/D | Modulation of nucleic acid binding affinity | Tumor promoting | Direct protein regulation | CRC | N/D | MS identification/K→  R/in vivo model | Chen  et al.^104^ |
| NSUN2  (Q08J23) | K692 | N/D | N/D | Regulation of protein stability | Tumor promoting | Direct protein regulation | PDAC | N/D | MS identification/K→  R/in vivo model | Huang et al.^135^ |
| NSUN2  (Q08J23) | K508 | NAA10 | N/D | Modulation of enzymatic activity | Tumor promoting | Indirect transcriptional regulation | GC | N/D | MS identification/K→  R/in vivo model/clinical samples | Niu  et al.^161^ |
| NUDT21  (O43809) | K23 | AARS1 | HDAC2 | Protein-Protein interactions | Tumor promoting | Direct protein regulation | ESCC | N/D | MS identification/K→  R/in vivo model/clinical samples | Lin  et al.^59^ |
| NUSAP1  (Q9BXS6) | K34 | P300 | HDAC | Regulation of protein stability | Tumor promoting | Direct protein regulation | PDAC | N/D | MS identification/K→  A/in vivo model | Chen  et al.^37^ |
| p53  (P04637) | K120, K139 | AARS1 | N/D | Modulation of nucleic acid binding affinity/Regulation of protein stability/Regulation of subcellular localization | Tumor promoting | Direct protein regulation/Indirect transcriptional regulation | BC/Glioma | Chemotherapy | MS identification/K→  R/in vivo model/clinical samples | Zong  et al.^57^ |
| PARP1  (P09874) | K373–524 | N/D | N/D | Modulation of enzymatic activity | Tumor promoting | Direct protein regulation | LC | N/D | in vivo model | Wang  et al.^156^ |
| PARP1  (P09874) | K654 | N/D | SIRT2 | Modulation of enzymatic activity | Tumor promoting | Direct protein regulation | ESCC | Chemotherapy | MS identification/K→R | Peng  et al.^69^ |
| PARP1  (P09874) | K498, K505, K506, K508, K518, K521, K524 | N/D | N/D | Modulation of enzymatic activity | Tumor promoting | Direct protein regulation | LICA | N/D | K→R | Zhou  et al.^144^ |
| PCBP2  (Q15366) | K115 | N/D | N/D | Regulation of protein stability/Protein-Protein interactions | Tumor promoting | Direct protein regulation | GC | Chemotherapy | K→R/in vivo model/clinical samples | Zhao  et al.^158^ |
| PD-L1  (Q9NZQ7) | N/D | N/D | N/D | Regulation of protein stability | Tumor promoting | Direct protein regulation | GC | Immunotherapy | in vivo model/clinical samples/in vivo model | Hu  et al.^177^ |
| PD-L1  (Q9NZQ7) | K280 | AARS1 | N/D | Regulation of protein stability/Protein-Protein interactions | Tumor promoting | Direct protein regulation | NSCLC | Immunotherapy | MS identification/K→  R/in vivo model/clinical samples | Liang  et al.^178^ |
| PD-L1  (Q9NZQ7) | K271 | GCN5 | N/D | Regulation of protein stability | Tumor promoting | Direct protein regulation | N/D | Immunotherapy | MS identification/K→  R/in vivo model/clinical samples | Tong  et al.^87^ |
| PFKM  (P08237) | K678 | N/D | N/D | Modulation of enzymatic activity | Tumor promoting | Direct protein regulation | EC | Chemotherapy/Immunotherapy | MS identification/K→  R/in vivo model/clinical samples | Wang  et al.^98^ |
| PFKP  (Q01813) | K392 | N/D | N/D | Regulation of protein stability/Modulation of enzymatic activity | Tumor promoting | Direct protein regulation | OC | N/D | K→R/in vivo model/clinical samples | Mi  et al.^76^ |
| PIK3CB  (P42338) | K415 | N/D | N/D | Modulation of enzymatic activity/Protein-Protein interactions | Tumor promoting | Direct protein regulation | LUAD | N/D | N/DMS identification/K→  R/in vivo model | Rao  et al.^115^ |
| PKM2  (P14618) | K115 | N/D | N/D | Modulation of enzymatic activity/Regulation of subcellular localization | Tumor promoting | Direct protein regulation/Indirect transcriptional regulation | CcRCC | N/D | MS identification/K→  R/K→Q/in vivo model/clinical samples | Guo  et al.^97^ |
| PKM2  (P14618) | K206 | AARS1 | N/D | Regulation of subcellular localization/Protein-Protein interactions | Tumor promoting | Direct protein regulation/Indirect transcriptional regulation | CRC | Targeted | MS identification/K→  R/in vivo model/clinical samples | Li  et al.^141^ |
| PKM2  (P14618) | K433 | P300 | N/D | Modulation of enzymatic activity/Regulation of subcellular localization | Tumor promoting | Direct protein regulation/Indirect transcriptional regulation | BLCA | N/D | MS identification/K→  R/in vivo model | Jin  et al.^164^ |
| POLR2A  (P24928) | K1125 | N/D | N/D | Regulation of protein stability/Protein-Protein interactions | Tumor promoting | Direct protein regulation/Indirect transcriptional regulation | TNBC | N/D | K→R/in vivo model/clinical samples | Yuan  et al.^145^ |
| PRDX1  (Q06830) | K67 | N/D | N/D | Protein-Protein interactions/Regulation of subcellular localization | Tumor promoting | Indirect transcriptional regulation | HCC | Targeted | MS identification/K→  R/in vivo model/clinical samples | Yang  et al.^159^ |
| PRMT1  (Q99873) | K134, K145 | KAT7 | HDAC8 | Modulation of enzymatic activity/Protein-Protein interactions | Tumor promoting | Direct protein regulation | TNBC/NSCLC | N/D | MS identification/K→  R/in vivo model/clinical samples | Zhou  et al.^146^ |
| PRMT5  (O14744) | K240 | N/D | N/D | Protein-Protein interactions/Regulation of protein stability | Tumor promoting | Indirect transcriptional regulation/RNA modification | CRC | N/D | MS identification/K→  R/in vivo model/clinical samples | Qu  et al.^163^ |
| PSMD14  (O00487) | K100 | N/D | N/D | Regulation of protein stability/Protein-Protein interactions | Tumor promoting | Direct protein regulation | ICC | Immunotherapy | MS identification/K→  R/in vivo model/clinical samples | Cai  et al.^99^ |
| PTBP1  (P26599) | K13, K436 | ND | SIRT1 | Regulation of protein stability /Modulation of nucleic acid binding affinity | Tumor promoting | Direct protein regulation | Glioma | N/D | MS identification/K→  R/in vivo model/clinical samples | Zhou  et al.^68^ |
| Rab7A  (P51149) | K21, K31, K32 | P300 | N/D | Modulation of enzymatic activity/Protein-Protein interactions/Regulation of subcellular localization | Tumor promoting | Direct protein regulation | HCC | N/D | MS identification/K→  R/in vivo model/clinical samples | Jiang  et al.^190^ |
| Rac2  (P15153) | N/D | P300 | N/D | N/D | Tumor promoting | Direct protein regulation | LUAD | N/D | in vivo model/clinical samples/Co-IP | Yi  et al.^181^ |
| RAD51  (Q06609) | K40, K73 | GCN5 | N/D | N/D | Tumor promoting | Direct protein regulation | OC | Chemotherapy | MS identification/K→  R/in vivo model/clinical samples | Sun  et al.^152^ |
| RBM15  (Q96T37) | K850 | N/D | HDAC3 | Regulation of protein stability/Protein-Protein interactions | Tumor promoting | Direct protein regulation/RNA modification | NSCLC | N/D | K→R/in vivo model/clinical samples | Zhao  et al.^64^ |
| RCC2  (Q9P258) | K124 | KAT2A | N/D | Protein-Protein interactions/Regulation of subcellular localization | Tumor promoting | RNA modification | BC | N/D | MS identification/K→  R/in vivo model/clinical samples | Zheng  et al.^50^ |
| RET  (P07949) | K549 | P300 | N/D | Regulation of protein stability | Tumor promoting | Direct protein regulation | HCC | N/D | MS identification/K→  R/in vivo model/clinical samples | Zhang  et al.^119^ |
| RHOA  (P61586) | K118, K162 | PCAF | HDAC3 | Modulation of enzymatic activity | Tumor promoting | Direct protein regulation | BC | N/D | MS identification/K→  R/K→Q/in vivo model/clinical samples | Ma  et al.^118^ |
| RPA1  (P27694) | K88, K163, K167, K267 | HAT1 | N/D | Modulation of nucleic acid binding affinity | Tumor promoting | Direct protein regulation | LUAD | Radiotherapy | MS identification/K→  R/in vivo model/clinical samples | He  et al.^48^ |
| SHMT2  (P34897) | N/D | N/D | N/D | Regulation of protein stability/Protein-Protein interactions | Tumor promoting | Direct protein regulation | ESCA | N/D | in vivo model/clinical samples | Qiao  et al.^100^ |
| SLC26A3  (P40879) | K536 | P300,CBP | N/D | Regulation of protein stability/Protein-Protein interactions/Regulation of subcellular localization | Tumor promoting | Direct protein regulation | CRC | Chemotherapy | MS identification/K→  R/in vivo model/clinical samples | Chen  et al.^122^ |
| Snail1  (O95863) | N/D | N/D | N/D | Regulation of subcellular localization | Tumor promoting | Indirect transcriptional regulation | PC | N/D | in vivo model/clinical samples | Zhao  et al.^129^ |
| SNRPA  (P09012) | K123 | AARS1 | N/D | Protein-Protein interactions/Regulation of subcellular localization | Tumor promoting | Indirect transcriptional regulation | PCA | Targeted | MS identification/K→  R/in vivo model/clinical samples | Zhao  et al.^197^ |
| SOX9  (P48436) | N/D | N/D | N/D | N/D | Tumor promoting | N/D | NSCLC | N/D | CO-IP/WB/in vivo model | Yan  et al.^124^ |
| SP1  (P08047) | K19 | N/D | N/D | Modulation of nucleic acid binding affinity | Tumor promoting | Indirect transcriptional regulation | EC | N/D | MS identification/K→  R/in vivo model/clinical samples | Ma  et al.^132^ |
| SPRING  (Q9H741) | K82 | KAT7 | N/D | Regulation of subcellular localization/Protein-Protein interactions | Tumor promoting | Indirect transcriptional regulation | ICC | Chemotherapy/Immunotherapy | MS identification/K→  R/in vivo model/clinical samples | Zhu  et al.^162^ |
| SPTAN1  (Q13813) | K1952, K1957 | AARS1 | HDAC1 | Regulation of subcellular localization/Protein-Protein interactions | Tumor promoting | Indirect transcriptional regulation | HCC | Immunotherapy | MS identification/K→  R/in vivo model/clinical samples | Wang  et al.^143^ |
| STAT1  (P42224) | K193 | AARS1 | N/D | Protein-Protein interactions | Tumor promoting | Indirect transcriptional regulation | CRC | Immunotherapy | MS identification/K→R/in vivo model/clinical samples | Du  et al.^93^ |
| SUMO2  (P61956) | K11 | AARS1 | HDAC1 | Protein-Protein interactions | Tumor promoting | Direct protein regulation | LUAD | Chemotherapy/Immunotherapy | MS identification/K→  R/in vivo model/clinical samples | Shan  et al.^56^ |
| TFEB  (P19484) | K91 | P300 | N/D | Regulation of protein stability/Protein-Protein interactions | Tumor promoting | Indirect transcriptional regulation | PC | N/D | MS identification/K→  R/K→Q/in vivo model/clinical samples | Huang et al.^166^ |
| TPX2  (Q9ULW0) | K249 | CBP | HDAC1 | Protein-Protein interactions | Tumor promoting | Direct protein regulation | HCC | N/D | MS identification/K→  R/in vivo model/clinical samples | Liu  et al.^121^ |
| TRIM29  (Q14134) | N/D | N/D | N/D | Protein-Protein interactions/Regulation of protein stability | Tumor promoting | Direct protein regulation | GC | Chemotherapy | in vivo model/clinical samples/in vivo model | Huimin et al.^130^ |
| VE-cadherin  (P33151) | N/D | LDHA | N/D | N/D | Tumor promoting | Direct protein regulation | GBM | N/D | in vivo model/clinical samples | Zhang  et al.^189^ |
| VEGFR2  (P35968) | N/D | LDHA | N/D | N/D | Tumor promoting | Direct protein regulation | GBM | N/D | in vivo model/clinical samples | Zhang  et al.^189^ |
| VIRMA  (Q69YN4) | K1713 | AARS2 | N/D | Modulation of enzymatic activity | Tumor promoting | RNA modification | CRC | N/D | MS identification/K→  R/in vivo model/clinical samples | Tan  et al.^187^ |
| Vps34  (Q8NEB9) | K356, K781 | KAT5, TIP60 | N/D | Modulation of enzymatic activity/Protein-Protein interactions | Tumor promoting | Direct protein regulation | LC/GC | N/D | MS identification/K→  R/in vivo model/clinical samples | Jia  et al.^167^ |
| XLF  (Q9H9Q4) | K288 | GCN5 | SIRT1 | Protein-Protein interactions/Regulation of subcellular localization | Tumor promoting | Direct protein regulation | CRC | Chemotherapy/Radiotherapy | MS identification/K→  R/in vivo model/clinical samples | Jin  et al.^49^ |
| XRCC1  (P18887) | K247 | N/D | N/D | Protein-Protein interactions/Regulation of subcellular localization | Tumor promoting | Direct protein regulation | GBM | Chemotherapy/Radiotherapy | MS identification/K→  R/in vivo model/clinical samples | Li  et al.^82^ |
| YAP  (P46937) | K90 | AARS1 | N/D | Regulation of subcellular localization/ Modulation of nucleic acid binding affinity | Tumor promoting | Indirect transcriptional regulation | HCC | Targeted | MS identification/K→  R/in vivo model/clinical samples | Wang  et al.^92^ |
| YAP  (P46937) | K102 | N/D | N/D | Regulation of subcellular localization | Tumor promoting | Direct protein regulation/Indirect transcriptional regulation | HCC | N/D | K→R/in vivo model | Wei  et al.^131^ |
| YBX1  (P67809) | K92 | P300 | SIRT2 | Modulation of nucleic acid binding affinity | Tumor promoting | Direct protein regulation/RNA modification | CRC | N/D | MS identification/K→  R/in vivo model/clinical samples | Chen  et al.^70^ |
| YTHDC1  (Q96MU7) | K82 | P300 | HDAC3 | Protein-Protein interactions/Regulation of protein stability | Tumor promoting | RNA modification | RCC | Targeted | MS identification/K→  R/K→Q/in vivo model/clinical samples | Dai  et al.^45^ |
| YTHDC1  (Q96MU7) | K82 | AARS1 | N/D | Regulation of protein stability/Protein-Protein interactions | Tumor promoting | Indirect transcriptional regulation | BLCA | Targeted | MS identification/K→  R/in vivo model/clinical samples | Xing  et al.^60^ |
| YTHDF3  (Q7Z739) | K544, K548 | AARS2 | SIRT2, SIRT3 | Regulation of protein stability/Protein-Protein interactions | Tumor promoting | RNA modification | BLCA | Chemotherapy | MS identification/K→  R/in vivo model/clinical samples | Yu  et al.^9^ |
| YY1  (P25490) | K183 | N/D | N/D | Protein-Protein interactions | Tumor promoting | Indirect transcriptional regulation | GBC | N/D | K→R/in vivo model/clinical samples | Wu  et al.^125^ |
| ZMIZ1  (Q9ULJ6) | K843 | N/D | N/D | Regulation of protein stability/Protein-Protein interactions | Tumor promoting | Indirect transcriptional regulation | BC | Targeted | MS identification/K→  R/in vivo model/clinical samples | Liu  et al.^128^ |
| ARHGDIB  (P52566) | K47, K50 | P300 | HDAC2 | Protein-Protein interactions | Tumor suppressing | Direct protein regulation | BLCA | Chemotherapy | MS identification/K→  R/in vivo model/clinical samples | Xu  et al.^63^ |
| CA3  (P07451) | K36 | N/D | SQLE | Regulation of protein stability | Tumor suppressing | Direct protein regulation | HCC | N/D | MS identification/K→  R/in vivo model/clinical samples | Yan  et al.^173^ |
| CNPY3  (Q9BT09) | K215, K224 | N/D | SIRT1 | Regulation of subcellular localization | Tumor suppressing | Direct protein regulation | PCA | N/D | MS identification | Zhang  et al.^67^ |
| DHX15  (O43143) | K17 | N/D | HDAC2 | Protein-Protein interactions | Tumor suppressing | Direct protein regulation/RNA modification | BLCA | Chemotherapy | MS identification/K→  R/in vivo model/clinical samples | Xu  et al.^61^ |
| METTL16  (Q86W50) | K229 | AARS1, AARS2 | SIRT2 | Modulation of enzymatic activity | Tumor suppressing | Indirect transcriptional regulation | GC | N/D | MS identification/K→  R/in vivo model/clinical samples | Sun  et al.^21^ |
| PD-L1  (Q9NZQ7) | K189 | P300 | HDAC2 | Protein-Protein interactions/Regulation of subcellular localization | Tumor suppressing | Indirect transcriptional regulation | LICA | N/D | K→R/in vivo model/clinical samples | Wang  et al.^62^ |
| PFKP  (Q01813) | K688 | N/D | N/D | Modulation of enzymatic activity | Tumor suppressing | Direct protein regulation | CRC | N/D | MS identification/in vivo model/clinical samples | Cheng  et al.^7^ |
| PPP1R11  (O60927) | K59 | N/D | N/D | Regulation of protein stability/Modulation of enzymatic activity | Tumor suppressing | Direct protein regulation | CRC | Chemotherapy | MS identification/K→  R/in vivo model/clinical samples | Xia  et al.^192^ |
| PPP1R14B  (Q96C90) | K140 | N/D | N/D | Regulation of protein stability | Tumor suppressing | Direct protein regulation | CECA | N/D | MS identification/K→R | He  et al.^147^ |
| RIG-I  (O95786) | K518, K657 | PCAF | N/D | Protein-Protein interactions/Regulation of subcellular localization | Tumor suppressing | Direct protein regulation | LUAD | Chemotherapy | MS identification/K→  R/in vivo model/clinical samples | Li  et al.^91^ |
| ALDOA  (P04075) | K147 | N/D | N/D | Modulation of enzymatic activity/Regulation of protein stability | N/D | Direct protein regulation | N/D | N/D | MS identification/K→R | Wan  et al.^33^ |
| ATRX  (P46100) | K438, K584, K1099 | N/D | N/D | Protein-Protein interactions | N/D | Indirect transcriptional regulation | N/D | N/D | MS identification | Bao  et al.^10^ |

ALL, Acute lymphoblastic leukemia. BC, Breast cancer. BLCA, Bladder cancer. CC, Colon cancer. CECA, Cervical cancer. CRC, Colorectal cancer. ccRCC, Clear cell renal cell carcinoma. EC, Endometrial cancer. ESCA, Esophageal cancer. ESCC, Esophageal squamous cancer. GBM, Glioblastoma. ‌ GBC, Gallbladder carcinoma. GC, Gastric cancer. HCC, Hepatocellular carcinoma. ICC, Intrahepatic cholangiocarcinoma. LC, Lung cancer. LICA, Liver cancer. LSCC, Laryngeal squamous cell carcinoma. LUAD, Lung adenocarcinoma. MEL, Melanoma. NB, Neuroblastoma. NSCLC, Non-Small cell lung cancer. OC, Ovarian cancer. PCA, Prostatic cancer. PC, Pancreatic cancer. PDAC, Pancreatic ductal adenocarcinoma. RCC, Renal cell carcinoma. TNBC, Triple-negative breast cancer.
