# Supplementary material for: Non-histone protein L-lactylation in cancer: a metabolic switch for immune evasion and therapeutic resistance
Source: Mol Cancer. 2026 Aug 1;25:259. doi: 10.1186/s12943-026-02759-7 (PMC13721481; doi:10.1186/s12943-026-02759-7)
Supplement: Supplementary file 2 [file 12943_2026_2759_MOESM2_ESM.docx]

**Table 2 Lactylation events in drug resistance treatment**

|  | **Specific drugs /**  **treatment plans** | **Lactylated substrate** | **Modification site** | **Catalyzing enzyme** | **Categories of drug resistance mechanisms** | **Type of cancer** | **References** |
| --- | --- | --- | --- | --- | --- | --- | --- |
| Chemoth-erapy and Radioth-erapy | Cisplatin, PARP inhibitors (olaparib) | MRE11 | K673 | CBP | Enhanced DNA repair | Colorectal cancer, ovarian cancer, gastric cancer, etc. | Chen et al.^44^ |
|  | Cisplatin | NBS1 | K388 | TIP60 | Enhanced DNA repair | Colorectal cancer, gastric cancer, lung cancer, ovarian cancer, etc. | Chen et al.^17^ |
|  | Platinum-based drugs | XLF | K288 | GCN5 | Enhanced DNA repair | Colorectal cancer, gastric cancer, etc. | Jin et al.^49^ |
|  | cis-platinum | ARHGDIB | K47/K50 | P300 | Enhanced DNA repair | Bladder cancer | Xu et al.^63^ |
|  | cis-platinum | DHX15 | K17 | HDAC2 | Survival signal maintenance | Bladder cancer | Xu et al.^61^ |
|  | Temozolomide | XRCC1 | K247 | N/D | Enhanced DNA repair | Glioblastoma | Li et al.^82^ |
|  | Platinum-based drugs | PARP1 | K654 | N/D | Enhanced DNA repair | Various types of cancer | Wang et al.^156^ |
|  | Radiotherapy | Ku70/Ku80 | multi-site | - | Enhanced DNA repair | Lung adenocarcinoma | He et al.^11^ |
|  | Radiotherapy | RPA1 | K88/K163/K167/K267 | HAT1 | Enhanced DNA repair | Lung adenocarcinoma, glioblastoma | Zhao et al.^47^  He et al.^48^ |
|  | 5-Fluorouracil, etc. | eEF1A2 | K408 | KAT8 | Survival signal maintenance | Colorectal cancer | Xie et al.^85^ |
|  | Anthracycline drugs | BLM | K24 | AARS1 | Enhanced DNA repair | Various types of cancer | Li et al.^18^ |
| Targeted therapy | Androgen deprivation therapy (ADT), enzalutamide | SNRPA | K123 | AARS1 | Survival signal maintenance | Prostate cancer | Zhao et al.^197^ |
|  | EGFR-TKI (Osimertinib) | ENO1 | K89 | p300/CBP | Metabolic compensation | Non-small cell lung cancer | Gan et al.^19^ |
|  | EGFR-TKI (Osimertinib) | Mic60 | K282 | - | Metabolic compensation | Non-small cell lung cancer | Zheng et al.^113^ |
|  | Lenvatinib | ABCF1 | K430 | p300 | Survival signal maintenance | Prostate cancer | Hong et al.^36^ |
|  | Lenvatinib | AKR1B10 | K173 | AARS1 | Metabolic compensation | Liver cancer | Liu et al.^53^ |
|  | Lenvatinib | IGF2BP3 | K76 | PARK7 | Metabolic compensation | Liver cancer | Lu et al.^112^ |
|  | Regorafenib | PRDX1 | K67 | - | Survival signal maintenance | Colorectal cancer | Yang et al.^159^ |
|  | Tamoxifen | ZMIZ1 | K843 | - | Survival signal maintenance | Breast cancer | Liu et al.^128^ |
|  | Enzalutamide, ADT | hnRNPA1 | K179 | p300 | Survival signal maintenance | Prostate cancer | Sun et al.^39^ |
|  | ADT, Enzalutamide | SNRPA | K123 | AARS1 | Survival signal maintenance | Prostate cancer | Zhao et al.^197^ |
|  | BRAF/MEK inhibitors | LSD1 | K503 | - | Survival signal maintenance | Melanoma | Li et al.^88^ |
| Immuno-therapy | PD-1 inhibitors | APOC2 | K70 | P300 | Immunosuppression | Non-small cell lung cancer | Chen et al.^182^ |
|  | PD-1/PD-L1 inhibitors | PD-L1 | K280/K271 | AARS1/GCN5 | Immunosuppression | Non-small cell lung cancer, various types of cancer | Tong et al.^87^  Liang et al.^178^ |
|  | PD-1/PD-L1 inhibitors | MOESIN | K72 | - | Immunosuppression | Various types of cancers (such as liver cancer, lung cancer, colorectal cancer, etc.) | Gu et al.^179^ |
|  | PD-1 inhibitors | METTL3 | K281/K345 | P300 | Immunosuppression | Colorectal cancer, liver cancer, etc. | Xiong et al.^41^ |
|  | CAR-T therapy | CD44 | K158 | AARS1 | Immunosuppression | Colorectal cancer | Yang et al.^86^ |
|  | PD-1 inhibitors | ENSA | K63 | EP300 | Immunosuppression | Pancreatic cancer | Sun et al.^198^ |
|  | PD-1/PD-L1 inhibitors | STAT1 | K193 | AARS1 | Immunosuppression | Various types of cancer | Du et al.^93^ |
|  | PD-1 inhibitors | cGAS | K21 | - | Immunosuppression | Various types of cancer | Rao et al.^115^ |
