# Supplementary material for: Non-histone protein L-lactylation in cancer: a metabolic switch for immune evasion and therapeutic resistance
Source: Mol Cancer. 2026 Aug 1;25:259. doi: 10.1186/s12943-026-02759-7 (PMC13721481; doi:10.1186/s12943-026-02759-7)
Supplement: Supplementary file 3 [file 12943_2026_2759_MOESM3_ESM.docx]

**Table 3 Comparison of Pharmacological Intervention Strategies Targeting Lactylation**

| Intervention level | Target class | Representative targets | Representative tools / compounds | Mechanism of action | Development stage | Key advantages | Major limitations | Representative evidence |
| --- | --- | --- | --- | --- | --- | --- | --- | --- |
| Upstream | Lactate-producing enzyme | LDHA | Fx11, Gen-140, NCI-006... | Inhibition of pyruvate–lactate conversion, reducing intracellular lactate supply for lactylation | Preclinical | Direct metabolic targeting; multiple inhibitors available | Systemic metabolic toxicity; limited event selectivity | Zhang et al.^212^  Cai et al.^213^  Peng et al.^214^ |
|  | Lactate transporter | MCT1 / MCT4 | AZD3965, syrosingopine... | Blockade of lactate transport disrupting tumor–stromal metabolic symbiosis | Preclinical | Disrupts tumour metabolic ecosystem | Broad metabolic effects in normal tissues | Xu et al.^215^  Puri et al.^216^  Wang et al.^211^  Singh et al.^217^ |
| Midstream | Writers | p300, CBP | C646, A-485... | Inhibition of lactyltransferase activity, reducing histone and non-histone lactylation | Preclinical | Targets modification machinery directly | Broad substrate spectrum; global epigenetic effects | Gan et al.^19^  Wang et al.^38^  Jiang et al.^224^ |
|  |  | AARS1, AARS2 | Alaninol... | Interference with lactyl-tRNA formation affecting lactylation reactions | Conceptual | Potentially closer to lactylation reaction | Risk of interfering with protein translation | Liu et al.^53^ |
|  | Erasers | HDAC1–3, SIRT3 | TSA... | Modulation of delactylation processes | Conceptual | Reversible regulation of modification levels | Strong pleiotropy across multiple PTMs | Yang et al.^65^ |
| Downstream | Functional lactylation events | Lactylated substrate proteins | Interfering peptides | Disruption of lactylation-dependent protein interactions or functional interfaces | Conceptual | Event-specific targeting; highest theoretical precision | Structural knowledge required; delivery challenges | Singh et al.^217^  Liu et al.^230^ |
|  |  |  | PROTAC-based concepts | Selective degradation of oncogenic lactylated proteins | Conceptual | Potential modification-state-specific targeting | Lack of lactylation-specific binders; major technical barriers | Sun et al.^231^  Chang et al.^232^ |
| Combination | Upstream + Standard Anti-tumor Therapy | LDHA/MCT + DNA repair proteins | Oxamate + Cisplatin, ENO1 antibody + Osimertinib, AZD3965 + Anti-PD-1 inhibitorchemotherapy | Blocks upstream lactate production/transport, inhibits treatment-induced lactylation cascade, reverses lactylation-mediated multi-modal resistance with standard anti-tumor therapy. | Preclinical | Synergy with established therapies | Therapeutic window and metabolic toxicity | Gan et al.^19^  Zeng et al.^102^  Gu et al.^179^ |
|  | Midstream + Standard Anti-tumor Therapy | p300 + oncogenic signalling pathways | A-485 + Enzalutamide, Chidamide + Cisplatin | Targets lactylation writer/eraser enzymes, blocks resistance pathways, synergizes with standard therapy to boost therapeutic efficacy. | Preclinical | Potential resistance-delaying strategy | Global epigenetic effects | Sun et al.^39^  He et al.^42^  Liu et al.^53^ |
|  | Downstream + Standard Anti-tumor Therapy | Specific Lactylated Substrate + Radiotherapy/Anti-Angiogenic Therapy | Saikosaponin D + Radiotherapy, AARS1 inhibitor + Bevacizumab | Selectively blocks pathogenic lactylation function, precisely targets core drug resistance pathways, and enables site-specific sensitization when combined with standard therapy. | Preclinical | Precisely targets resistance drivers, minimal normal tissue impact, strong standard therapy synergy, low off-target risk | Insufficient in vivo causal validation of core targets; lack of clinically available specific compounds | Li et al.^43^  Li et al.^141^ |
